# Transcriptomic and epigenomic profiling of young and aged spermatogonial stem cells reveals molecular targets regulating differentiation

**DOI:** 10.1371/journal.pgen.1009369

**Published:** 2021-07-08

**Authors:** Jinyue Liao, Hoi Ching Suen, Alfred Chun Shui Luk, Lele Yang, Annie Wing Tung Lee, Huayu Qi, Tin-Lap Lee

**Affiliations:** 1 Developmental and Regenerative Biology Program, School of Biomedical Sciences, Faculty of Medicine, The Chinese University of Hong Kong, Shatin, Hong Kong SAR, China; 2 Guangzhou Regenerative Medicine and Health Bioland Laboratory, Guangzhou Institutes of Biomedicine and Health, Guangzhou, China; UMass Medical School, UNITED STATES

## Abstract

Spermatogonial stem cells (SSC), the foundation of spermatogenesis and male fertility, possess lifelong self-renewal activity. Aging leads to the decline in stem cell function and increased risk of paternal age-related genetic diseases. In the present study, we performed a comparative genomic analysis of mouse SSC-enriched undifferentiated spermatogonia (Oct4-GFP+/KIT-) and differentiating progenitors (Oct4-GFP+/KIT+) isolated from young and aged testes. Our transcriptome data revealed enormous complexity of expressed coding and non-coding RNAs and alternative splicing regulation during SSC differentiation. Further comparison between young and aged undifferentiated spermatogonia suggested these differentiation programs were affected by aging. We identified aberrant expression of genes associated with meiosis and TGF-β signaling, alteration in alternative splicing regulation and differential expression of specific lncRNAs such as *Fendrr*. Epigenetic profiling revealed reduced H3K27me3 deposition at numerous pro-differentiation genes during SSC differentiation as well as aberrant H3K27me3 distribution at genes in Wnt and TGF-β signaling upon aging. Finally, aged undifferentiated spermatogonia exhibited gene body hypomethylation, which is accompanied by an elevated 5hmC level. We believe this in-depth molecular analysis will serve as a reference for future analysis of SSC aging.

## Author summary

Sperms are derived from a primitive population of undifferentiated spermatogonia called spermatogonial stem cells (SSCs), which lay the foundation of male fertility. It is widely known that the aging process results in decline in SSC function and quantity. However, the molecular mechanisms and gene expression changes underlying SSC differentiation and aging remain elusive. Here, we revealed the alterations in gene expression programs of both coding and non-coding RNAs, and alternative splicing regulation during SSC aging and differentiation by profiling the transcriptomes of SSC-enriched undifferentiated spermatogonia in mice. For instance, we found long non-coding RNA *Fendrr* is highly expressed in undifferentiated cells, suggesting its role in maintaining stem cell function. We further explored the changes at epigenetic level by profiling histone modifications and DNA methylation patterns, which revealed distinct distribution patterns of histone modification H3K27me3 in Wnt and TGF-β signaling upon aging. We also found that aged undifferentiated spermatogonia exhibited a decrease in average 5mC methylation level in gene bodies accompanied by an increase in 5hmC level, which is similar to the observations in humans. Therefore, we believe our work will serve as a reference for future investigation of human reproductive aging, and particularly the mechanisms of SSC aging.

## Introduction

In recent decades, there has been a remarkable delay in the paternal age of reproduction across high-income countries. This phenomenon leads to an increase in infertility rate. One area that contributes to age-related infertility is age-associated decline in spermatogonial stem cells. Spermatogonial stem cells (SSCs) are stem cells in the male germline which form the foundation of male fertility. Animal studies have shown that SSCs undergo an age-associated decline in function. For example, there is a dramatic decrease in the quantity of SSCs in aged mouse testes, which causes testicular regression or atrophy and an increased abundance of Sertoli cell-only tubules [1–3]. In contrast to non-germline stem cells, dysregulation of the SSC genome could cause significant health impacts in the offspring. For instance, animal aging models and human cohort studies revealed an increase in *de novo* mutations in germ cells and mature sperms as males enter advanced age. Such changes may lead to developing complex diseases in the offspring [4]. Aberrant epigenetic changes in SSC have also been indicated in transgenerational genetic diseases [5,6]. Therefore, studying the role of aging in deterioration of SSC function is crucial in the understanding of the aging-associated fertility decline and the impact of delayed parental age on the health of offspring.

Current data suggest that both stem cell intrinsic defects and alterations in microenvironment are implicated in the age-dependent decline of functional germ cell reserves [2,7,8]. Transcriptome studies indicated altered gene expression in aged SSCs, affecting functional pathways such as DNA damage responses, mitosis, and oxidative stress [9]. Highly dynamic and coordinated epigenetic processes in SSC are also vital for the fertility and health of future generations [10]. For example, several histone deacetylases (HDACs) are downregulated as adult SSCs differentiate or age [11]. Globally decreased activity of the PRC2 complex was observed in the aging of SSCs [12]. Several recent genomic profiling studies have attempted to determine the landscape of epigenetic regulation in SSC [13–19]. However, the understanding of how aberrant gene expression patterns, epigenetic alteration, and their interaction underlie SSC aging is limited.

The maintenance of SSCs is tightly coordinated through transcriptional and epigenetic programs. In this study, we investigated how aging contributes to the deterioration of SSC function from the perspective of transcriptional and epigenetic regulations in SSC-enriched undifferentiated spermatogonia. We first established the transcriptional dynamics during SSC differentiation and delineated the stem cell intrinsic molecular program underlying the age-dependent changes in SSC function. We further established global histone modification maps and identified the relationship between the epigenetic programming and gene expression signatures associated with spermatogonial differentiation and aging. In addition, we mapped the dynamics of 5mC and 5hmC at single-base resolution in aged SSC-enriched spermatogonia and uncovered aging-associated alterations in DNA modification. At last, we examined the impact of aging on chromatin accessibility. Taken together, our study will aid in better understanding of fundamental processes in SSC aging.

## Results

### Isolation and transcriptome profiling of undifferentiated and differentiating spermatogonia from adult testis

**Fig 1:**
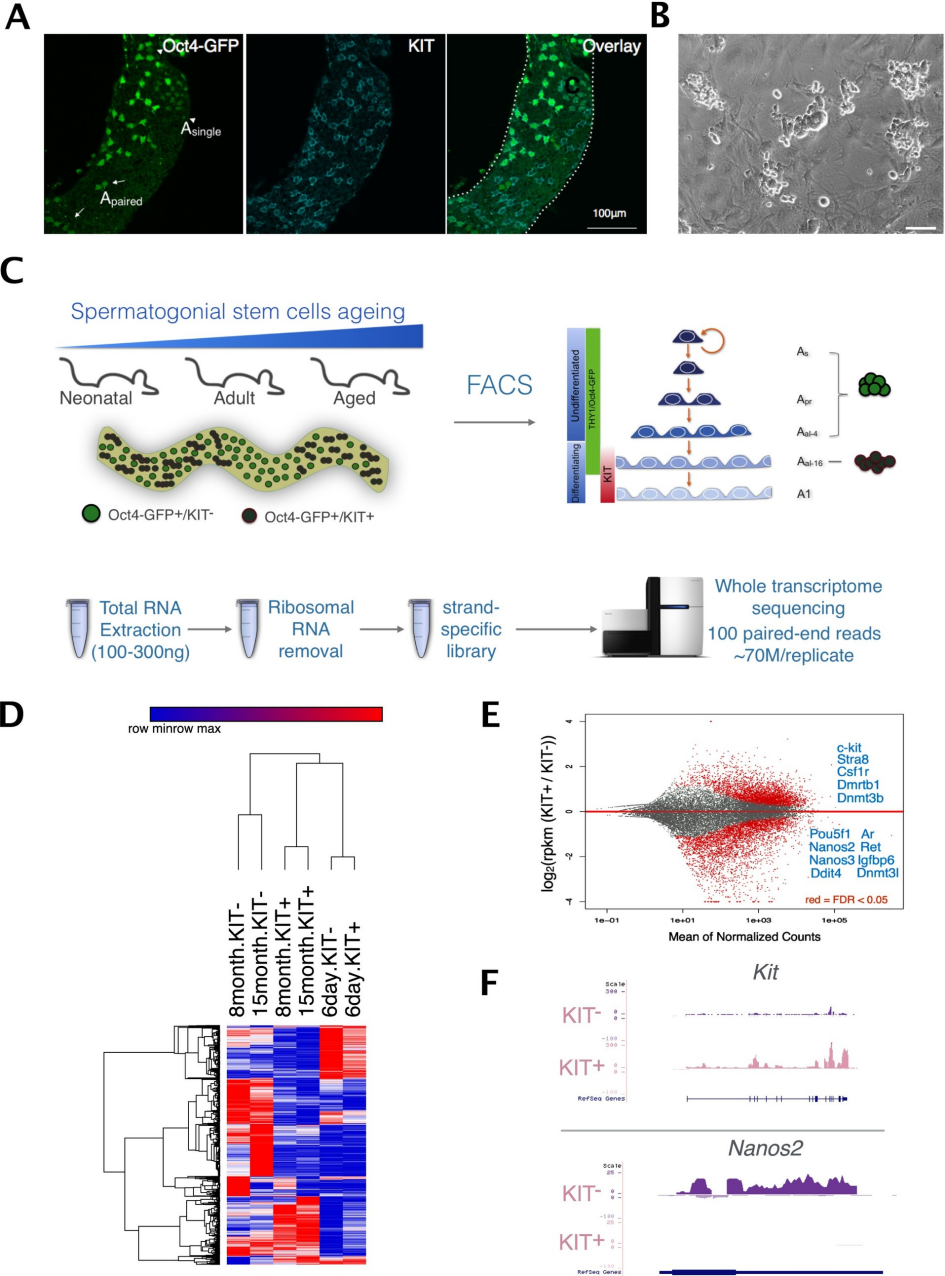
Gene expression dynamics in SSCs during the KIT transition and aging. (A) Whole-mount immunostaining showing the distribution of Oct4-GFP+ cells in adult testis. (B) Oct4-GFP+/KIT- cells isolated from adult testis can be maintained in vitro. (C) The schematic diagram showing the design of the sample collection and RNA-Seq analysis. (D) Heatmap showing global gene expression profiles of SSC populations differ significantly at different ages. (E) Differential gene expression between KIT- and KIT+ SSC populations in adult mice. Red dots represent differentially expressed genes with the statistical significance of FDR<0.05. Selected genes important for spermatogenesis in each fraction are shown in blue. (F) Genome browser view of the most significant differential gene expression, *Nanos2* and *Kit* in KIT- and KIT+ population respectively.

The common cell surface markers used for spermatogonia isolation are also expressed in other testicular cell types to some extent and genes expressed at high levels in contaminating cells can impact RNA-seq profile interpretations [20]. To overcome this problem, we utilized Oct4-GFP transgenic mice to avoid the contamination of other cell types. We found that GFP was solely expressed in undifferentiated (PLZF+) and differentiating (KIT+) spermatogonia, but not in spermatocytes (SYCP3+) or Sertoli cells (SOX9+) (S1 Fig). Notably, Oct4-GFP+/KIT- cells are mainly As (single) spermatogonia or Apr (paired) spermatogonia in 3-month-old adult testis through whole-mount immunostaining (Fig 1A). In addition, FACS-sorted Oct4-GFP+/KIT- cells from adult testis possessed the capacity for in vitro proliferation and colony formation (Fig 1B). Therefore, Oct4-GFP+/KIT- cell population is enriched for SSC and its characteristics can serve as a proxy for SSC. Based on these, we separated SSC-enriched undifferentiated spermatogonia (Oct4-GFP+/KIT-) and differentiating spermatogonia (Oct4-GFP+/KIT+) using FACS from neonatal (PND5.5), adult (6–8 months) and aged mice (15–18 months), which were subjected to RNA-Seq analysis (Fig 1C). Gene expression profiles of KIT- and KIT+ cells are well separated from each other in adult spermatogonia regardless of age, reflecting that they acquired more drastic transcription dynamics upon differentiation compared to the neonatal stage (Figs 1D and S2). In contrast, neonatal KIT- and KIT+ cells clustered closely together, indicating that early germ cells share similar gene expression properties despite different differentiation status (Figs 1D and S2). In addition, we found considerable regulatory differences between neonatal and adult spermatogonia.

### Identification of genes and pathways associated with the maintenance and aging of adult undifferentiated spermatogonia

We then compared KIT- and KIT+ cells with special focus on the adult stage. The genes with higher expression level in adult KIT- comprised the genes essential for SSC self-renewal and maintenance such as *Oct4*, *Ddit4*, *Ret* and *Nanos2* [21–24]. Genes facilitating SSC differentiation were upregulated in adult KIT+ cells, such as *Stra8* and *Dnmt3b* (Fig 1E and 1F). IPA analysis revealed downregulation of the Mouse Embryonic Stem Cell Pluripotency pathway during differentiation (S3 Fig). These results were in line with the known regulators of SSC. Besides known essential genes for SSC self-renewal such as *Oct4* and *Id4* in this pathway, the Wnt signaling component Frizzled receptor 2 (*Fzd2*) is also upregulated in KIT- cells. *Fzd2* has been reported as a marker for crypt base columnar (CBC) stem cells [25] and may have a role in SSC maintenance (S3 Fig). These results further supported that KIT- cells were enriched of cell populations with higher self-renewal capacity.

**Fig 2:**
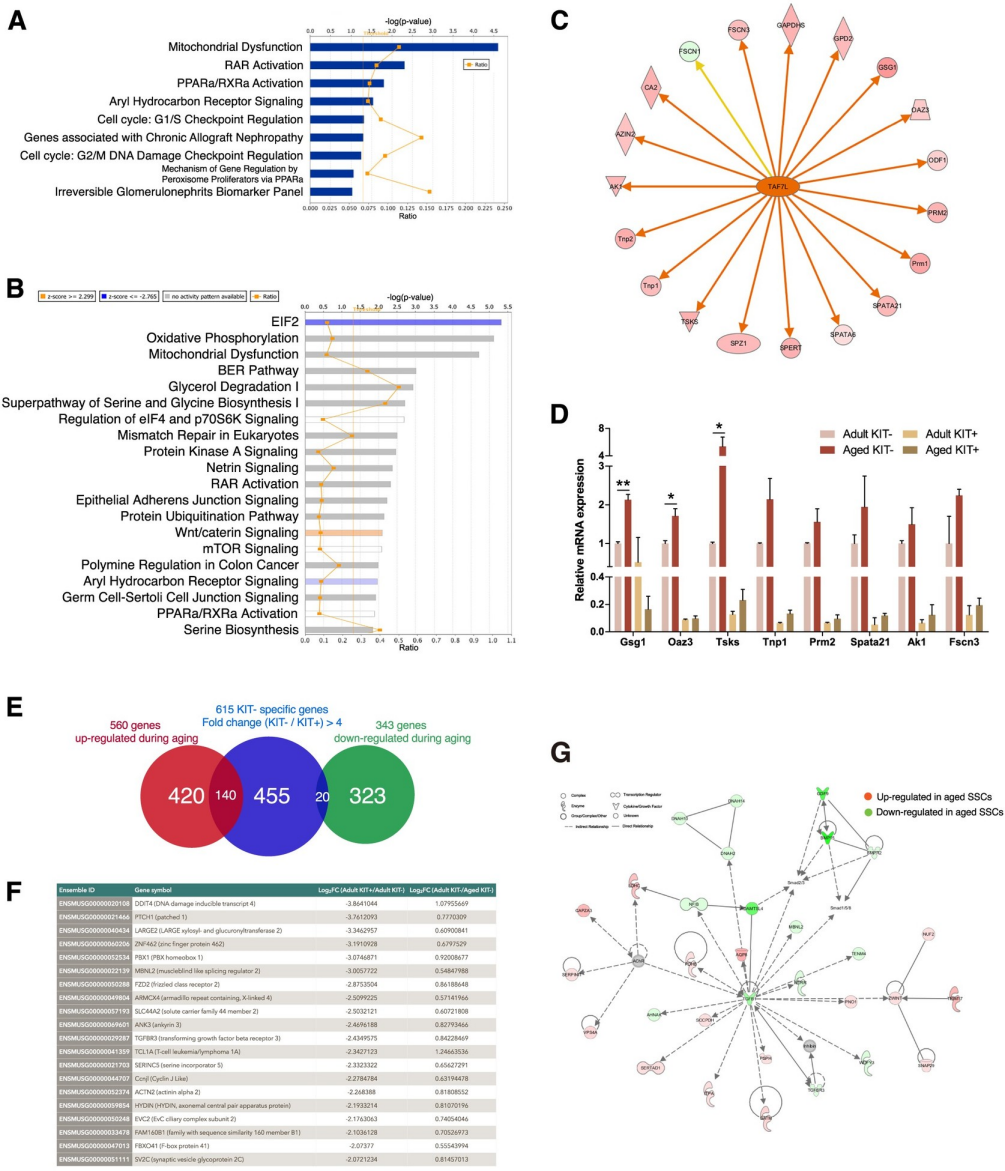
Genes and pathways associated with the maintenance and aging of adult SSCs. (A) Ingenuity pathway analysis (IPA) gene ontology analysis on the differentially expressed genes between adult and aged SSC showing Top toxicity target lists identified. (B) Top 20 canonical pathways as revealed by Ingenuity pathway analysis (IPA) gene ontology analysis on the differentially expressed genes between adult and aged SSC. (C) Inference of *Taf7l* regulation from the aging SSC transcriptome. Upstream regulator analysis by Ingenuity pathway analysis (IPA). Data illustrate *Taf7l* as “activated” in aged cells in upstream regulator analysis. Genes in red were greater expressed in aged cells compared with young adults, whereas genes in green were lower expressed. An orange line indicates predicted upregulation, whereas the yellow line indicates expression being contradictory to the prediction. (D) RT-PCR analysis of expression of Taf7l downstream targets showing greater expression in IPA in aged cells compared with adult cells (n = 2, unpaired t-test). Error bars are plotted with SD. (E) Aging-associated differential expression of the KIT- preferential genes. Venn diagram of the overlap between genes differentially expressed upon aging and genes highly enriched in KIT- cells. (F) The full list of the KIT- preferential genes showing down-regulation upon aging. (G) Inference of TGF-β signaling from the aging SSC transcriptome. Shown is the network from Ingenuity Pathway Analysis (IPA) consisting of TGF- β1 regulators (e.g., TGFB1, TGFBR3, and BMP5) and the subset of SSC aging differentially expressed genes. Symbolic representations of genes, expression changes, and regulatory relationships are shown on the top.

We then investigated the altered gene expression upon aging in undifferentiated KIT- cells. IPA analysis revealed mitochondrial dysfunction and peroxisome proliferator-activated receptors (PPARs) activation as the “top targets of toxicity” as the effect of aging (Fig 2A). PPARs are important regulators in various age-associated pathophysiological processes related to energy metabolism and oxidative stress [26], and their deregulated activation might be the culprits for the SSC aging process. This is consistent with the recent finding showing decreased mitochondria numbers and expression of *Ppargc1a*, a co-activator of PPARs, in cell-intrinsic mode of SSC aging [12]. IPA canonical pathway analysis revealed the eIF2 pathway, a master regulator of cell adaptation to various forms of stress, was inhibited upon aging (Fig 2B), which could make SSC more susceptible to stress. Ribosome pathway was also altered, which is consistent with the previous finding that the rate of protein synthesis declines with age [27]. Therefore, our results suggested that many common signaling pathways contributing to aging can be recapitulated in aged SSCs.

We further uncovered changes related to several spermatogonia-specific pathways that might contribute to aging. Genes with decreased expression are involved in receptor binding, cell adhesion molecule binding, integrin binding, adhesion junction and focal adhesion, suggesting that the adhesion strength between SSCs and their niche is decreased upon aging and eventually affects their long-term self-renewal potential. Strikingly, we found the aberrant expression of a large number of genes associated with spermatogenesis. For example, increased expression of the known germ cell differentiation and meiosis markers such as *Sycp2*, *Sycp3* and *Stra8* were observed in aged undifferentiated spermatogonia. Consistent with this, we found that genes upregulated in the aged cells were significantly enriched for meiosis pathway and post-meiotic development such as sperm flagellum and spermatid differentiation. IPA analysis identified *Taf7l*, which regulates a group of genes related to meiosis, as the most significant upstream regulator responsible for transcriptional changes in aging SSCs, and is required for meiotic cell cycle progression in mouse spermatogenesis (Fig 2C) [28,29]. We further examined the gene expression of *Taf7l* downstream targets by qRT-PCR, and we found several genes were upregulated in aged cells, such as *Gsg1*, *Oaz3* and *Tsks* (Fig 2D). Moreover, a point mutation of human *TAF7L* is associated with infertility [30].

We then focused on the alteration of genes preferentially expressed in KIT- cells to ask how aging affects genes presumably important for SSC maintenance. Surprisingly, we only found 20 out of 615 (3%) KIT- preferential genes showed a declined trend during aging (Fig 2E). Among these genes, *Ddit4* is of particular interest because of its high expression, large fold-changes and strong specificity in KIT- cells (Fig 2F). *Ddit4* has been identified as a putative PLZF target and plays a critical role in the regulation of SSC self-renewal via inhibiting mTORC1 activity [22]. Other notable downregulated genes include: *Mbnl2*, a member of the muscleblind protein family that modulates alternative splicing of pre-mRNAs [31]; *Ptch1*, a component of hedgehog signaling which is linked with age-related diseases [32]; *Tcl1*, a known downstream target of *Oct4*. On the other hand, 140 of KIT- enriched genes (22%) increased upon aging (Fig 2E). GO analysis concurs with aforementioned results that they are mainly associated with the spermatogenesis-related function such as spermatid development, flagellated sperm motility, and fertilization.

Intriguingly, we performed network analysis using IPA to define the functional networks of aging associated genes and identify a top ranked network centered around the TGF-β pathway (Fig 2G), supporting the notion that irregularity of TGF-β signaling may contribute to the functional age-related declines that occur in stem cells with age [33].

### Alternative splicing regulation in the maintenance and aging of adult undifferentiated spermatogonia

**Fig 3:**
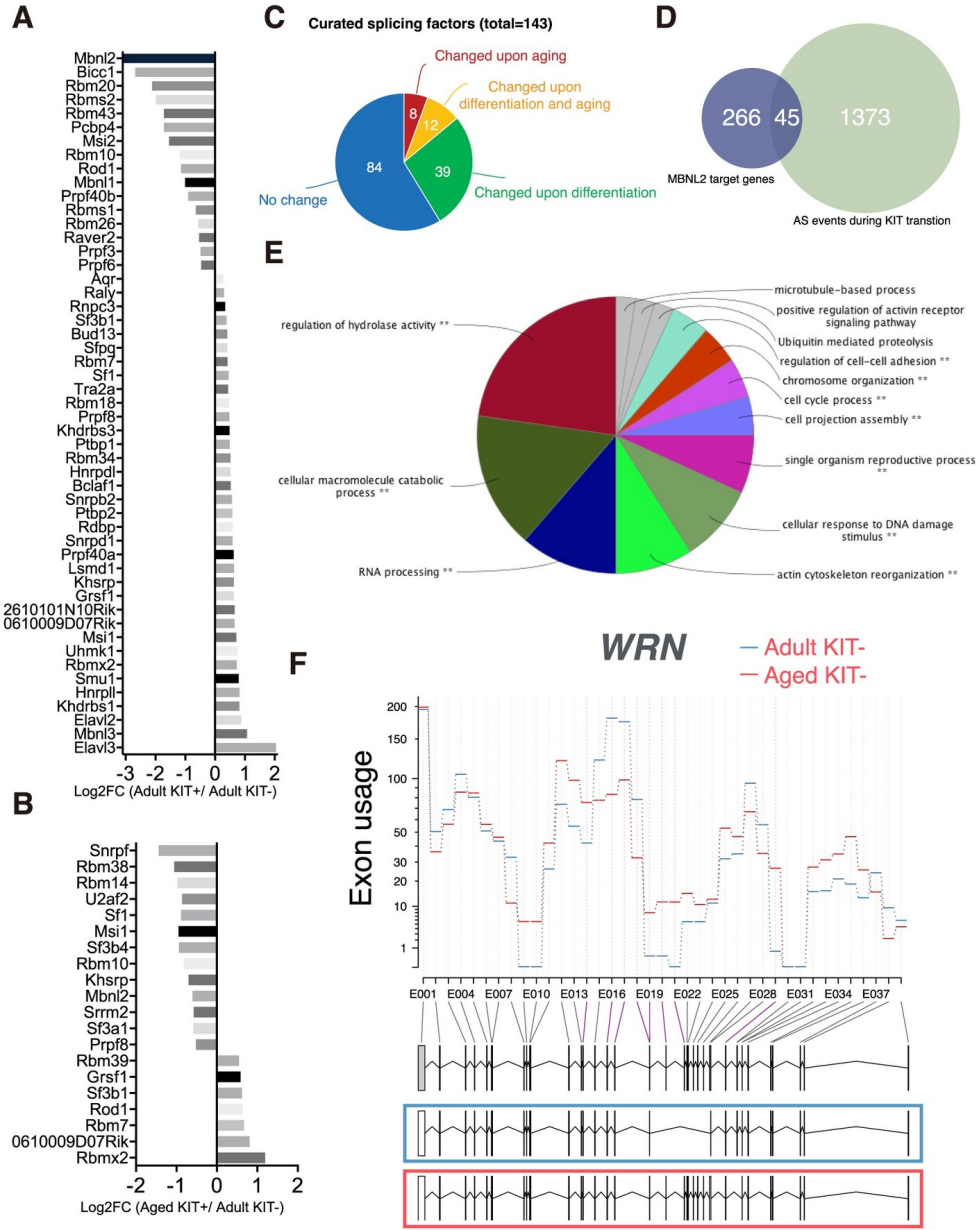
Post-transcriptional regulation during SSC differentiation and aging. (A-B) Column bar chart shows the fold change of splicing factors identified as differentially expressed during differentiation (A) and aging (B). (C) Pie chart showing the proportion of splicing factors whose expression levels were altered during SSC differentiation and aging. (D) Venn diagram of the overlap between MBNL2 target genes and differential alternative splicing events detected during differentiation. (E) GO enrichment analysis of the altered genes by comparing adult and aged undifferentiated SSCs. (F) The example of WRN showed differential exon usage upon aging, indicating regulated isoform expression level at different stages.

Recent results highlight that alternative splicing also contributed to cell- and species-specific differentiation [34,35]. Our analyses showed that splicing regulator *Mbnl2* is highly enriched in KIT- cells and displays aberrant expression upon aging (Fig 2F). GO analysis using both age-upregulated and age-downregulated genes revealed that the major pathways associated with older SSCs were mainly involved in the processing of the primary RNA transcripts (GO:0003723: RNA binding, p<7.026E-5 and p<3.731E-17, respectively). To comprehensively analyze the alteration in the splicing landscape, we monitored the expression of all known RNA splicing regulators expressed in spermatogonia (n = 143) during the KIT transition and aging. We found 51 and 20 regulators exhibited altered expression in KIT transition and during aging respectively (Fig 3A–3C). For example, we observed downregulation of both *Mbnl1* and *Mbnl2* encoding muscleblind proteins and the upregulation of *Elavl2*, *Elavl3*, and *Mbnl3* during KIT transition. We further validated the upregulation of splicing factors by qRT-PCR, which showed the significant increase of mRNA level in *Mbnl2*, *Bicc1* and *Rbm20* in KIT- cells compared with KIT+ cells (S4 Fig).

To monitor the splicing regulation, we analyzed the differential usage of exons between KIT- and KIT+ cells using DEXseq and found 1418 genes that showed evidence of differential exon usage. The alternative splicing events detected cover 45 out of 313 MBNL2 binding targets, which is consistent with the fact that MBNL2 is the most specific splicing factor enriched in KIT- cells (Fig 3D) [36]. On the other hand, genes with alternative splicing changes occurring during aging are associated with regulation of hydrolase activity, RNA processing, cellular response to DNA damage and cell cycle regulation (Fig 3E). We used ToppGene to survey the mouse phenotype database and found that mice with alteration in these genes shown phenotypes related to infertility such as small testis (p = 1.818E-5), decreased male germ cell number (p = 2.910E-5) and abnormal male germ cell morphology (p = 8.766E-5). Interestingly, the Werner syndrome protein gene (*WRN*), a member of RecQ family of DNA helicases, showed differential alternative splicing (Fig 3F). Mutations in the *WRN* gene give rise to Werner syndrome (WS) and the affected individuals exhibit features of accelerated aging [37].

These findings suggest transcript isoform regulation is an unexpectedly abundant regulatory mechanism in SSC differentiation and aging.

### Dynamic lncRNA expression during spermatogonial differentiation and aging

**Fig 4:**
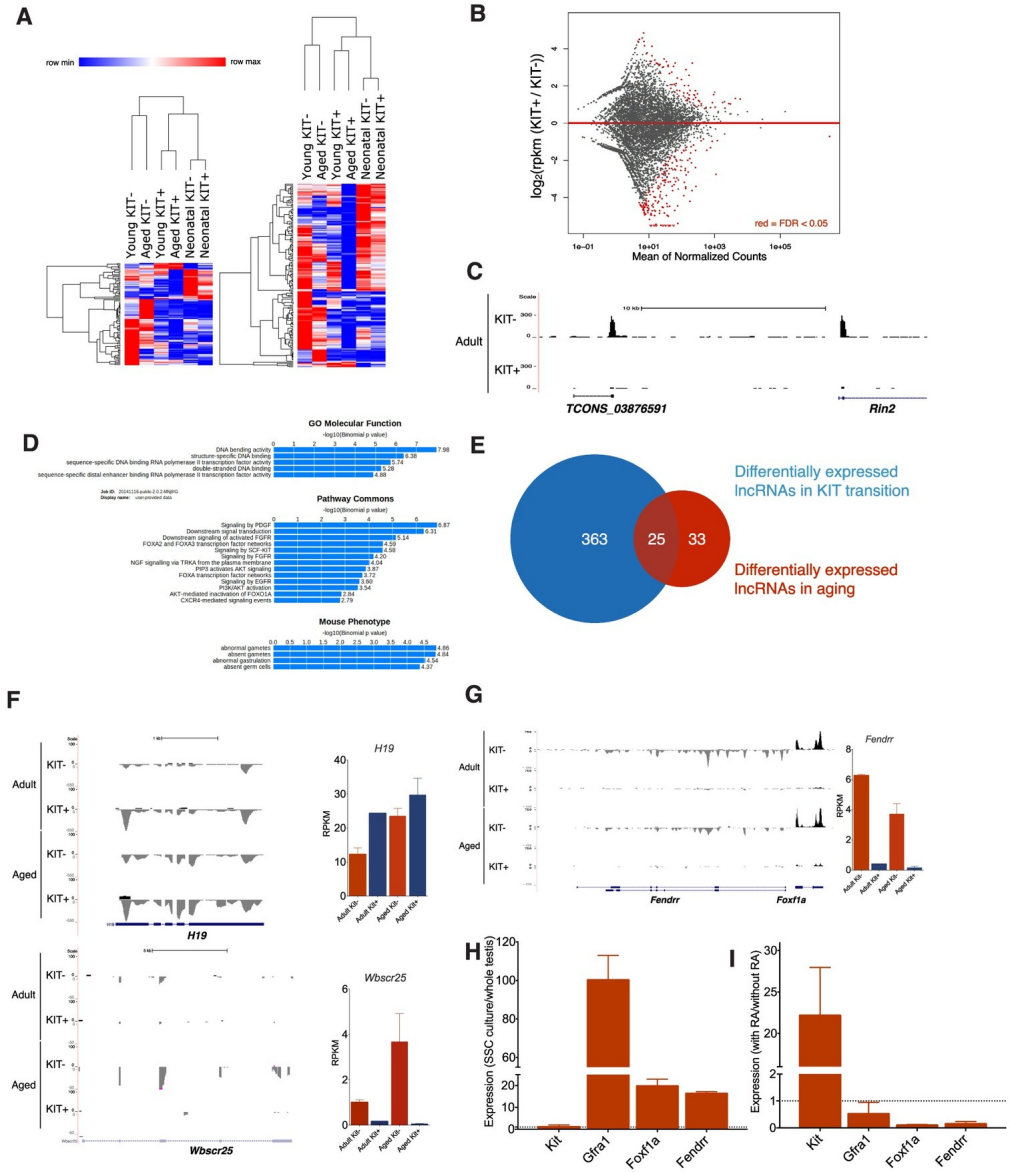
Expression of lncRNAs is tightly regulated during SSC differentiation and aging. (A) Heatmaps showing hierarchical clustering of differentially expressed transcripts of both known and novel lincRNA. (B) The MA plot shows the logarithm of the ratio between expression levels in KIT- and KIT+ populations versus the average expression of individual genes. The red dots depict the differentially expressed lncRNA (FDR< 0.05). (C) UCSC Genome Browser tracks for the KIT- cells specific novel lncRNA TCONS_03876591. (D) Functional prediction of lncRNAs regulated during differentiation by “Guilt-by-association” (GBA) analysis. (E) Venn diagram showing the number of lncRNAs with highly significant expression difference during KIT transition and aging (FDR<0.05) and 25 lncRNAs found commonly involved in both processes. (F) UCSC Genome Browser tracks for *H19* and *Wbscr25* with expression levels shown on the right. (G) UCSC Genome Browser tracks for *Fendrr* with expression levels shown on the right. (H) Relative expression levels of *Fendrr* and *Foxf1a* between SSC culture and whole testis (dash dot line: y = 1). (I) Expression level changes of *Fendrr* and *Foxf1a* after RA treatment (dash dot line: y = 1).

Recently, lncRNAs have been linked to stem cell self-renew and detrimental pathways regulating the aging process in skeletal muscle stem cells, hematopoietic stem cells and gut epithelium [38]. Therefore, we tested the hypothesis that the manifestations of aging in SSCs are associated with dysregulation of lncRNA expressions. We first employed the Ensembl annotation to provide a standardized and up-to-date analysis of lncRNA gene expression according to the biotype annotations. Out of the 3,506 genes annotated as “lincRNA”, “anti-sense” or “non-coding” (referred to as “lncRNA” for the remainder of this study), we detected 1,433 expressed at ≥ 1 FPKM. We subsequently established a bioinformatic pipeline for detecting novel lncRNAs (S1 Text and S5A and S5B Fig). S6 Fig showed several examples of the most abundant novel lncRNAs identified. Using DESeq2, we conducted the differential gene expression analysis and performed clustering using all differentially expressed lncRNAs. Our analysis showed that lncRNAs, similar to their coding counterparts, distinguished adult from neonatal, KIT- cells from KIT+ cells and young adult from aged adult samples (Fig 4A), suggesting that the expression of lncRNAs is also tightly regulated during SSC differentiation and aging.

Next, we characterized the expression of lncRNAs within the spermatogonial differentiation paradigm. We compared the adult KIT- and KIT+ samples and identified 388 lncRNAs displaying a significant change (FDR < 0.05) (Fig 4B). For example, TCONS_03876591 is a novel lincRNA that is specifically expressed in KIT- cells (Fig 4C). It is located near *Rin2*, which was also significantly differentially expressed between KIT- and KIT+ cells. Considering that lncRNAs have been shown to regulate proximal coding genes in *cis*, we searched for protein-coding genes 1000 kb upstream and downstream of the lncRNAs [39]. GO analysis of *cis* lncRNA targets revealed significant overrepresentation in terms involved in the regulation of gene expression, such as DNA bending activity, structure-specific DNA binding, sequence-specific DNA binding RNA polymerase II transcription factor activity and double-stranded DNA binding (Fig 4D), which is in line with the reported roles of lncRNA as transcriptional regulator. Pathway analysis showed that these *cis* target genes of lncRNAs were enriched in pathways that were related to spermatogenesis such as PDGF, FGF and SCF-KIT signaling pathways (Fig 4D). These findings suggested that lncRNAs might act on its neighbouring protein-coding genes in *cis* to regulate spermatogonial differentiation.

Next, we identified 58 lncRNAs with altered expression in the course of aging, 25 of which are also differentially expressed during differentiation (Fig 4E). For example, the lncRNA *H19* has been shown to play a role in growth, proliferation, cell cycle, apoptosis, and aging [40]. Enhanced expression of *H19* due to loss of imprinting of the *H19* locus was observed in normal human prostate tissues during aging. Consistent with this, we found the expression level of *H19* was elevated in aged undifferentiated spermatogonia (Fig 4F). Another candidate is *Wbscr25* (Williams Beuren syndrome chromosome region 25), which is highly enriched in KIT- cells and increased expression in aged cells (Fig 4F).

Among the known lncRNAs with significant expression changes in the process of KIT transition and aging, *Fendrr* (Fetal-lethal noncoding developmental regulatory RNA) caught our attention (Fig 4G). It displayed exclusive expression in KIT- cells, suggesting its potential role in stem cell maintenance. *Fendrr* is divergently transcribed from the transcription factor-coding gene *Foxf1a*, and they share a similar expression pattern. We speculated that it should be highly expressed in SSC cultures, but at low levels in the adult testis, where true stem cells are a rare subpopulation. Using RT-PCR, we confirmed the relative expression levels of *Fendrr* and its neighbouring gene *Foxf1a* were higher in SSC cultures than whole adult testis (Fig 4H). This lncRNA-protein gene pair also underwent significant changes in response to spermatogonial differentiation, with a decrease of almost 90% after 48 hours of RA treatment (Fig 4I).

### Decreased SSC differentiation is associated with decreased gene bivalency

**Fig 5:**
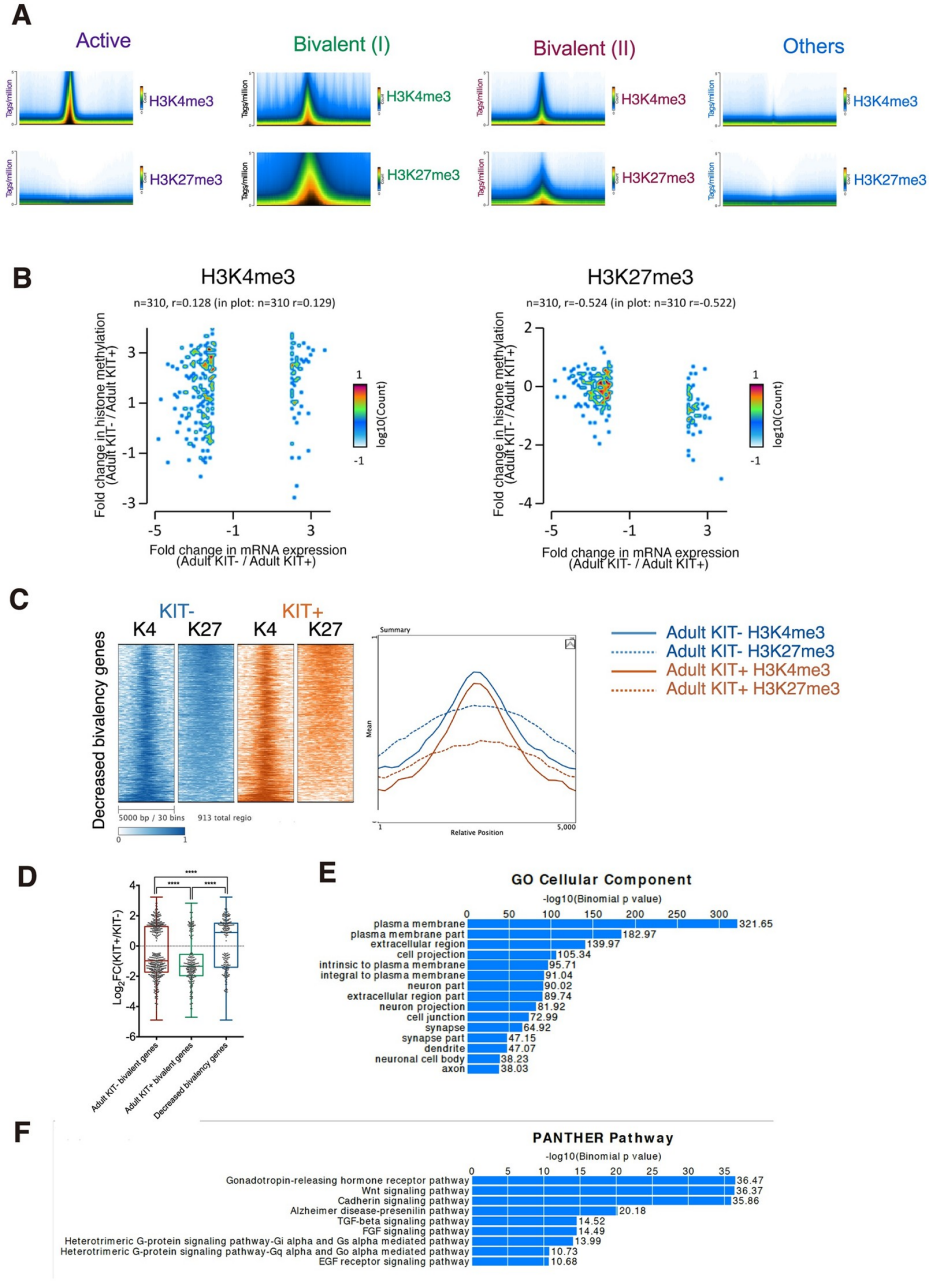
Gene bivalency on transcriptional regulation during the adult KIT transition. (A) The average profile plots for each category of promoter. (B) Negative correlation of H3K27me3 difference and gene expression fold change. (C) Heat maps (Left) and meta-plot (Right) of histone modifications across the 5 kb region centered at the TSSs of the genes with decreased bivalency during the KIT transition. (D) Box plots represent the spread of the gene expression changes as a result of the loss of bivalency during the adult KIT transition. The whiskers represent 5–95 percentile with the medians shown as horizontal lines. The p-values were calculated using unpaired, two-tailed t-test with 95% confidence. (E) Bar plot showing enrichment of biological processes for genes with decreased bivalency. (F) Bar plot showing enrichment of biological processes for genes associated with decreased H3K27me3 marks during SSC aging.

To investigate the epigenetic regulatory programs behind the transcriptional changes associated with SSC differentiation and aging, we first examined how histone modifications may regulate the differentiation of spermatogonia. We performed genome-wide analysis of H3K4me3 and H3K27me3 by ChIP-Seq and identified a set of bivalent promoters (Fig 5A). We found bivalent promoters were overrepresented in genes encoding cell–cell signaling molecules, developmental regulators, cell adhesion molecules and embryonic morphogenic proteins. Previous reports have shown that bivalent promoters are dynamically regulated during stem cell differentiation, but the role of bivalency in the course of SSC differentiation remains largely unknown [41,42]. Globally, we found a moderate inverse correlation between the change in the enrichment of H3K27me3 with the change in expression of differentially regulated genes during differentiation (Fig 5B). We then examined the number of genes associated with each promoter class in each cell type (S8 Fig). Strikingly, we found that less differentiated KIT- cells are associated with more genes belonging to the H3K27me3-high bivalent (I) class. Careful inspection of the promoters failed to be categorized into H3K27me3-high bivalent class revealed that they decrease the repressive H3K27me3 mark but maintain the active H3K4me3 mark, representing promoters revolving to H3K4me3 monovalency (Fig 5C). Unlike stable bivalent promoters which have a predisposition for downregulation during differentiation, resolving promoters were associated with a significant upregulation of gene expression during the differentiation of spermatogonia (Fig 5D). For example, induction of the differentiation related genes *Kit* and *Stra8* was accompanied by decreased H3K27me3 level during differentiation (S7A and S7B Fig). GO analysis showed that this set of bivalent genes are associated with plasma membrane and cell junction (Fig 5E). Our data thus show that while the genomic location of H3K27me3 is mostly conserved, the extent of gene bivalency differs quantitatively during KIT transition. This suggested the involvement of resolving bivalency in transcriptional activation in SSC differentiation, which is in line with ESC differentiation [43].

It has been suggested that PcG-mediated H3K27me3 alteration drives many age-related changes and is often dysregulated in human malignancies [44–46]. Therefore, we continued to examine the bivalent domain in aged undifferentiated spermatogonia. We directly compared the epigenetic states of RefSeq promoters in adult KIT- and aged KIT- cells and found the distributions of H3K4me3 and H3K27me3 were highly similar (S7C Fig). As a result, no significant correlation between changes in levels of mRNA and H3K4/K27me3 levels during aging were found (S7D Fig). We employed SICER to identify differentially histone methylation enrichment across the genome [47]. 7080 differentially enriched H3K27me3 peaks were identified between adult KIT- and aged KIT- cells, in which 1734 peaks have increased enrichment in aged cells and 5346 peaks with reduced enrichment (S9 Fig). GO analysis performed on the list of genes associated with decreased H3K27me3 mark during aging showed the enrichment for GnRH, Wnt/β-catenin, and TGF-β pathway (Fig 5F).

### Age-dependent 5mC and 5hmC alteration in spermatogonia

**Fig 6:**
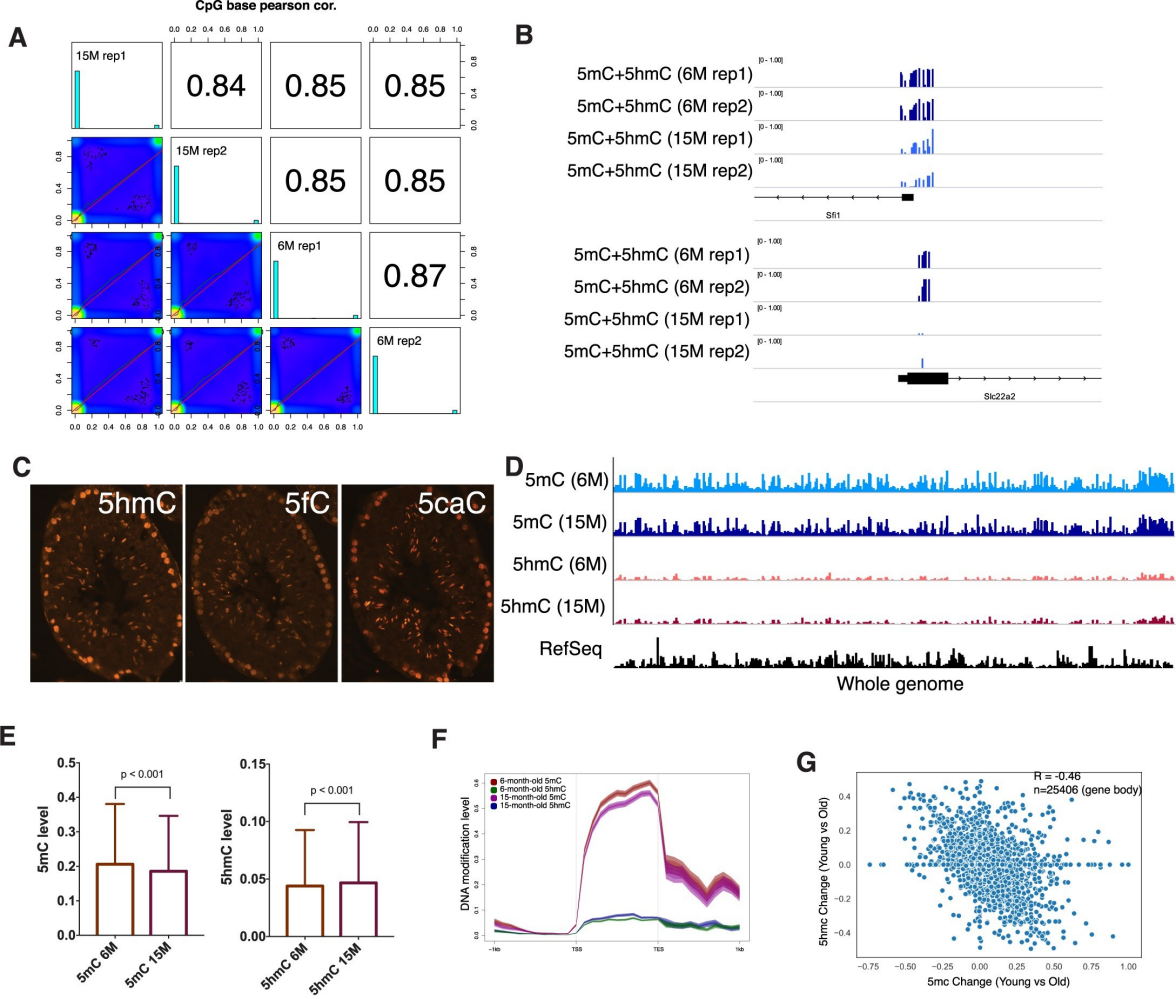
Effect of aging on 5mC and 5hmC in SSC. (A) Scatter plot and correlation of CpG methylation between RRBS methylomes. Scatter plots of percentage methylation values for each pair in four libraries. Numbers on the upper right corner denote pairwise Pearson’s correlation scores. The histograms on the diagonal are methylation distribution of CpG sites for each sample. (B) Examples of genomic RRBS profiles of differentially methylated promoters. (C) Representative images of testicular sections from adult mice showing immunofluorescent staining of 5hmC, 5fC, and 5caC, which were observed in cells residing on the basement membrane. (D) Genome browser view of 5mC and 5hmC distribution in all chromosomes. (E) The box plots show the average 5mC and 5hmC levels per bin (100kb) of the genome in young and aged SSCs. A pairwise comparison was performed using the independent sample t-test. (F) Metagene density profiles of 5mC and 5hmC for RefSeq transcripts showed levels of 5hmC are consistently higher in the aged SSCs than in the young SSCs along the length of the gene. (G) Scatterplot of “switching” between 5mC and 5hmC at gene bodies in aged SSC relative to young SSC.

We continued to investigate the effect of aging on DNA modification. We performed reduced-representation bisulfite sequencing (RRBS) and compared the DNA methylation level (5mC+5hmC) in young and aged undifferentiated spermatogonia. We found that the level of (5mC+5hmC) at individual CpG sites correlates well between the young and aged undifferentiated spermatogonia (Fig 6A). The 5mC differences at gene promoters are extremely rare and we only identified 3 genes, *Sfi1*, *Slc22a2*(*Oct2*) and *Gtf2f1* with a decrease in methylation larger than 30% (covered at least by 5 CpG sites) (Fig 6B). Interestingly, both *Sfi1* and *Slc22a2* have been implicated in the aging process, as the aging-associated methylation changes have been observed in several reports [48,49]. Many studies showed that 5hmC is linked with aging, but the role of 5hmC in SSC aging is largely unknown [50]. We first examined the active demethylation process in spermatogonia by immunostaining. Indeed, 5hmC and its oxidative products 5caC and 5fC are specifically located in the spermatogonia at the basal membrane (Fig 6C). Since conventional RRBS cannot distinguish 5mC and 5hmC, we employed oxRRBS which allowed us to measure 5mC and 5hmC separately [51]. In general, the levels of total 5hmC observed across the genome are approximately 10 folds lower than those of 5mC (Fig 6D). When we compared 5mC and 5hmC levels between the two groups, we noticed global 5mC was slightly but significantly decreased, while 5hmC level was increased in aged undifferentiated spermatogonia (Fig 6E). Plotting the average 5mC level across all genes revealed a global hypomethylation at the gene body in aged undifferentiated spermatogonia, akin to that observed in humans (Fig 6F) [52,53]. The reduced 5mC level at the gene body was accompanied by an increased 5hmC level (Fig 6F). Indeed, we found a very strong inverse correlation between reduction of average 5mC and increased level of 5hmC at gene bodies (Fig 6G). These results suggested that 5hmC-dependent active demethylation is associated with 5mC alteration during aging.

## Discussions

Understanding the underlying mechanisms conferring the functional decline in aged SSCs is one of the major questions to be addressed in reproductive biology. Early studies mainly focus on gene expression alteration during SSC aging [9,54]. These microarray analyses lacked the specificity of sequencing-based techniques and could not reveal gene isoform and novel transcript regulation. In this regard, our study provided a global view of the transcriptional and epigenetic programming that is associated with SSC normal differentiation and aging.

Our transcriptome profiles of spermatogonia provide a comprehensive transcriptomic map and reliable resource to study the molecular mechanisms of spermatogonial differentiation and the effect of aging. Aged undifferentiated spermatogonia apparently have a significantly altered gene expression signature compared to their younger counterparts. Pathway analysis revealed that many common age-related transcriptomic changes underlying both aging and the pathogenesis of multiple age-related diseases are also reflected in aged undifferentiated spermatogonia, illustrating the robustness and relevance of the current study. We also identified spermatogonia-specific aging pathways and our most striking observation is the aberrant expression of a large number of genes associated with meiosis pathway. Network analysis suggested that *Taf7l* might be one plausible candidate underlying this regulation. One possible explanation is that the mitotic and meiotic cell cycles should be kept in a tight control in SSC, but when germ cells are driven aberrantly from the mitotic into the meiotic cell cycle, their stem cell properties are lost.

A better understanding will not be possible without unravelling the essential mechanisms involved in the maintenance of SSCs and their differentiation in steady spermatogenesis. We have identified genes showing strong enrichment in KIT- cells, which might represent those important for SSC maintenance. We then examined whether there is an age-associated loss of their expression in mouse testes. Interestingly, we only identified a small percentage of genes enriched in undifferentiated spermatogonia with decreased expression during aging. Nevertheless, this analysis leads us to propose several SSC-specific factors including *Ddit4*, whose action mainly occurs through inhibition of the mTOR pathway [55]. mTOR can regulate stem cell function. Functional interaction between mTOR and PLZF is a critical rheostat for maintenance of SSC self-renewal, and this action is through *Ddit4* [22]. Hyperactive mTOR signaling has also been shown to have a plausible role involved in regulating aging in mammals [56]. Therefore, *Ddit4* might contribute to aging through modulating the mTOR pathway which regulates lifespan in multiple species. It has been demonstrated that *Ddit4* expression increased significantly in response to calorie restriction in both rats and mice, which is the only intervention known to extend lifespan [57,58]. Altogether, this finding points to *Ddit4* as a direct link between aging pathology and stem cell function decline in SSCs.

Epigenetic integrity is an essential element for maintaining normal stem cell function during aging [45]. Aged stem cells in mouse models feature aberrant expression of chromatin-modifying enzymes in various stem cell compartments [33,59]. Consistent with this, we also found that the expression of some chromatin modulators was ablated during aging. In addition, we present here a detailed examination of H3K27me3 modification in spermatogonial differentiation and aging. First, we found a specific set of genes encoding developmental regulators were bivalently marked with H3K4me3 and H3K27me3, which are significantly reduced during differentiation. This suggested that bivalent domains maintain developmental genes in a silent state in undifferentiated cells while keeping them poised for subsequent induction upon spermatogonial differentiation. Second, locus-specific alteration of H3K27me3 level was observed during aging. It has been reported that, however, the change in the level of H3K27me3 could not be linked to significant changes in mRNA levels of the associated genes. One possible explanation is that steady-state mRNA levels detectable by RNA-seq are not an accurate reflection of active transcription. Another possibility is that aging and age-related differential gene expression are multifactorial in nature and regulated by several other mechanisms. Previous characterization of SSC in vitro aging showed that H3K27me3 peaks were significantly decreased in the promoter regions of *Wnt7b* which resulted in *Wnt7b* up-regulation [12]. Similarly, we found genes associated with reduced H3K27me3 were related to common aging pathways such as Wnt and TGF-β pathways and their contribution to SSC aging has yet to be elucidated.

Current literature on how aging affects DNA methylation has been controversial. One possibility is that methylation levels in tissues have been measured instead of specific cell types. Our results analyzed the purified SSC-enriched undifferentiated spermatogonia and revealed there is a decrease in average 5mC level in gene bodies. Our results also indicated an age-related increased 5hmC level and the observed changes raised the possibility that 5hmC acts as an intermediate step of active DNA demethylation and contributed to hypomethylation. We further identified several promoters displaying changes in DNA methylation occur with age and may be functionally important. For example, one of the hypomethylated genes *Sfi1* is related to centrosome amplification, and interestingly, an age-related change in centrosome amplification was also identified in aged intestinal stem cells [49]. Moreover, upregulation of *Sfi1* probably causes other centrosome aberrations such as centrosome misorientation, which can lead to cell cycle arrest and consequently decline in spermatogenesis during aging [48].

In conclusion, our study indicates that the aging process in mouse SSCs is associated with transcriptome alteration, which is connected with epigenetic changes and may be mediated via histone modification, methylation and hydroxymethylation of DNA. We envision that our results could lay the groundwork for further exploration into the influence of epigenome dynamics in human reproductive aging.

## Materials and methods

### Ethics statement

All animal procedures were performed according to protocols approved by the Animal Experimentation Ethics Committee (AEEC) of the Chinese University of Hong Kong and following the Animals (Control of Experiments) Ordinance (Cap. 340) licensed from the Hong Kong Government Department of Health.

### Animals

Oct4-GFP transgenic mice (B6; CBA-Tg(Pou5f1-EGFP)2Mnn/J, Stock no.: 004654) were obtained from The Jackson Laboratory (Jackson Laboratory, Bar Harbor, ME, USA. Male transgenic mice at different ages (i.e., PND5.5 for neonatal group, 6-month-old for young group and 15-month-old for aged group) were employed in this study. Age and strain matched non-reporter mice i.e., C57BL/6 mice were used for cell purification using FACS sorter as negative control devoid of GFP expression.

### Cell isolation, sample preparation and sequencing

Testicular cells from adult and aged Oct4-GFP mice were isolated by a two-step enzymatic digestion. For RNA-seq experiment, total RNA was isolated from each cell fraction using the AllPrep DNA/RNA Mini kit (Qiagen, Valencia, CA, USA) and sequencing libraries were prepared with ribosomal RNA (rRNA) depletion using a Ribo-Zero Gold kit (Epicentre) followed by Apollo 324 NGS Library Prep System (WaferGen Biosystems, Fremont, CA, USA). Two biological replicates of the prepared libraries were sequenced on an Illumina HiSeq 2000 (Illumina, San Diego, CA) with 100 base pairs (bp) paired-end RNA-Seq reads. gDNA extracted from the same batch of cells are subjected to RRBS and oxRRBS experiments. 2x10^5^ freshly sorted Oct4-GFP+/KIT- or Oct4-GFP+/KIT+ cells were cross-linked with 1% formaldehyde for 10 min. ChIP experiments and ChIP-Seq libraries were prepared using Diagenode True MicroChIP kit according to the manufacturer’s protocol.

## Supporting information

S1 Fig Whole-mount immunostaining showing the distribution of Oct4-GFP+ cells in adult testis. Tubules of adult Oct4-GFP transgenic male mice are stained with SSC marker GFRA1, undifferentiated spermatogonia marker PLZF, spermatocyte marker SYCP3 and Sertoli cell marker SOX9.
(TIF)

S2 Fig Schematic representation of the Mouse Embryonic Stem Cell Pluripotency pathway highlighting the members identified as preferentially expressed in KIT- cells. Genes highlighted in red are identified as preferentially expressed in KIT- cells while genes labeled in green show higher expression in KIT+ samples. Noted that As marker *Id4* is identified in this analysis.
(TIF)

S3 Fig Principal component analysis showed that global gene expression profiles of SSC populations differ significantly at different ages. DEGs were subjected to PCA analysis to illustrate the relationships among transcriptomes. First, the first principal component (PC1) captured the differences between neonatal and adult germ cells, suggesting considerable regulatory differences in neonatal versus adult spermatogonia. Second, neonatal KIT- and KIT+ cells clustered closely together, indicating that early germ cells share gene expression properties that commonly define them in transcriptional space despite different differentiation status. Third, KIT- and KIT+ cells are well separated from each other in adult spermatogonia, in both normal and aged adults, reflecting that they acquired drastic transcription dynamics upon differentiation compared to the neonatal stage. Lastly, normal adult and aged cells are positioned far away from each other and KIT- cells are more separated compared to KIT+ cells, suggesting that age factor has a more pronounced effect on the undifferentiated spermatogonia than differentiating cells.
(TIF)

S4 Fig Quantitative assessment of splicing factors. qRT-PCR analysis of expression of splicing factors *Mbnl2*, *Bicc1* and *Rbm20* in adult KIT- cells compared with adult KIT+ cells (p < 0.05, n = 2, unpaired t-test). Error bars are plotted with SD.
(TIF)

S5 Fig Detection and quantification of lncRNAs. (A) Data sources for novel lncRNA identification. (B) A bioinformatics pipeline for discovery lncRNAs in SSC. See S1 Text Supplemental Methods session for details. Raw reads are first mapped onto the reference mouse genome. The initial assemblies are categorized by cuffcompare, compared with the combined gene annotations. The lncRScan program is performed to detect the novel lncRNAs from the high-quality assemblies according to multiple criteria.
(TIF)

S6 Fig UCSC Genome Browser tracks for the most expressed novel lncRNAs. (TIF)

S7 Fig Analysis of histone modification dynamics in SSC aging. (A) and (B) Genome browser representation of H3K4me3 and H3K27me3 modification at selected genes. (C) Quantitative comparisons of promoter read coverage (reads per kilobase) of each histone modification using 2.5-kb TSS centered bins. (D) Change in the enrichment of each histone modification bound to a gene promoter plotted against the change in expression of that gene using the set of differentially expressed genes during SSC aging.
(TIF)

S8 Fig H3K4me3 and H3K27me3 bivalent promoter methylation profiles in each cell population. (A) Clustering heatmaps demonstrate the distribution of H3K4me3 and H3K27me3 histone modifications at the promoters of the annotated transcripts in each cell population. (B) Comparison of the number of bivalent promoters in the different cell types according to clustering results showing in (A).
(TIF)

S9 Fig Genome browser representation of histone modification changes. Examples of individual genes showing the increased (A) or decreased (B) H3K27me3 enrichment.
(TIF)

S1 Text Supplemental Methods. (DOCX)
