# Supplementary figures and images for: Transcriptomic and epigenomic profiling of young and aged spermatogonial stem cells reveals molecular targets regulating differentiation
Source: PLoS Genet. 2021 Jul 8;17(7):e1009369. doi: 10.1371/journal.pgen.1009369 (PMC8291634; doi:10.1371/journal.pgen.1009369)

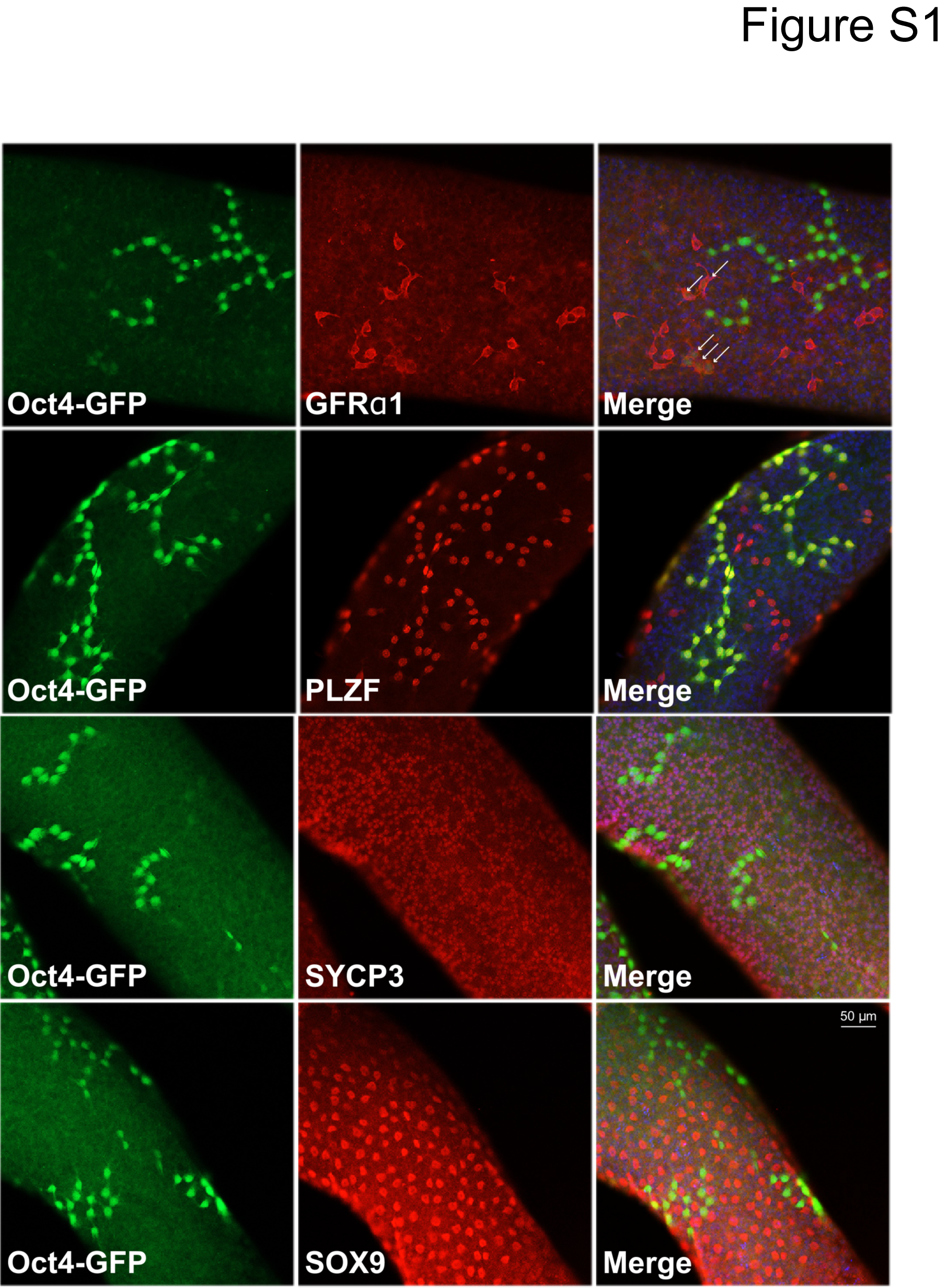

Supplement: S1 Fig — Tubules of adult Oct4-GFP transgenic male mice are stained with SSC marker GFRA1, undifferentiated spermatogonia marker PLZF, spermatocyte marker SYCP3 and Sertoli cell marker SOX9. (TIF) [file pgen.1009369.s001.tif]

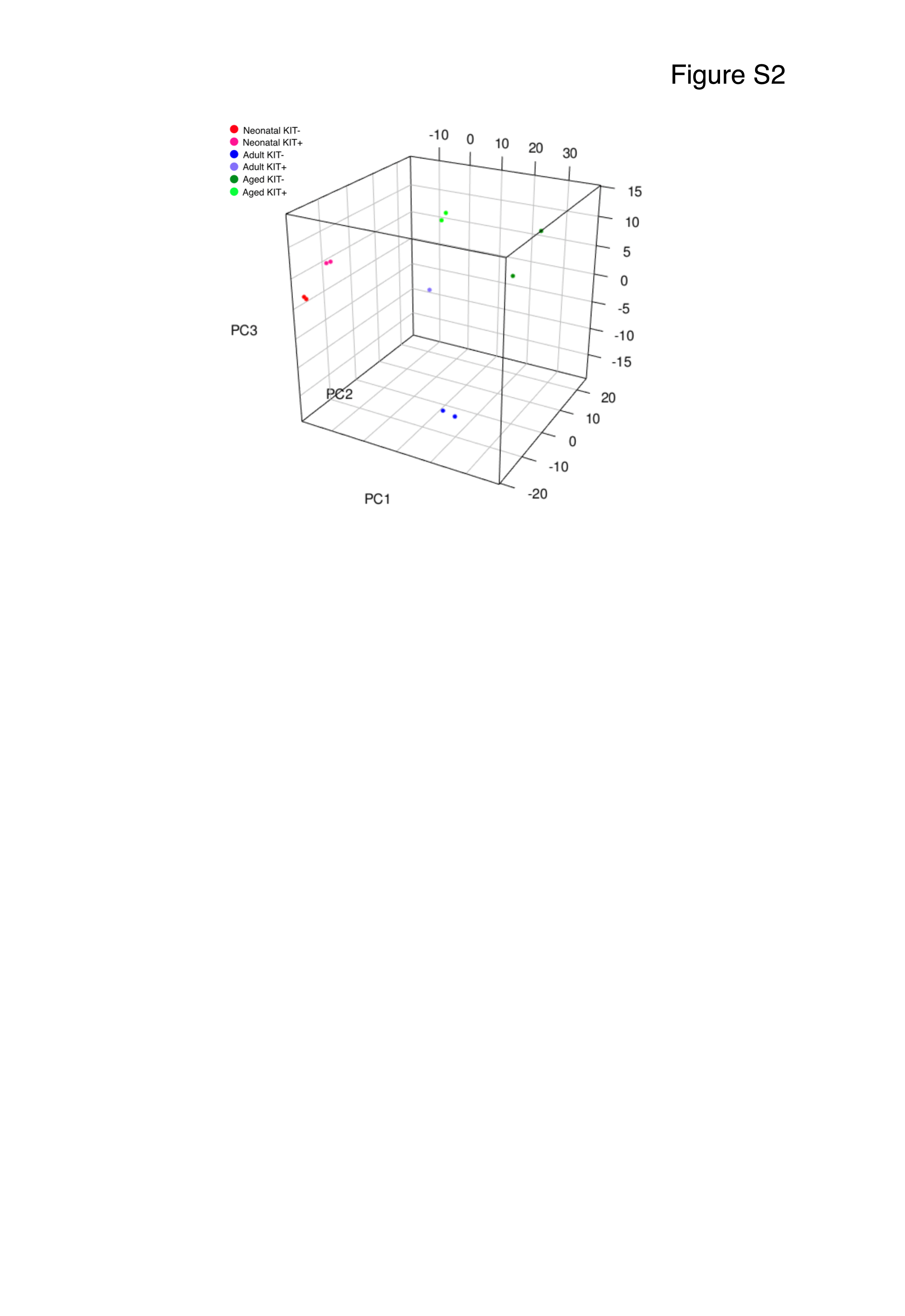

Supplement: S2 Fig — Genes highlighted in red are identified as preferentially expressed in KIT- cells while genes labeled in green show higher expression in KIT+ samples. Noted that As marker Id4 is identified in this analysis. (TIF) [file pgen.1009369.s002.tif]

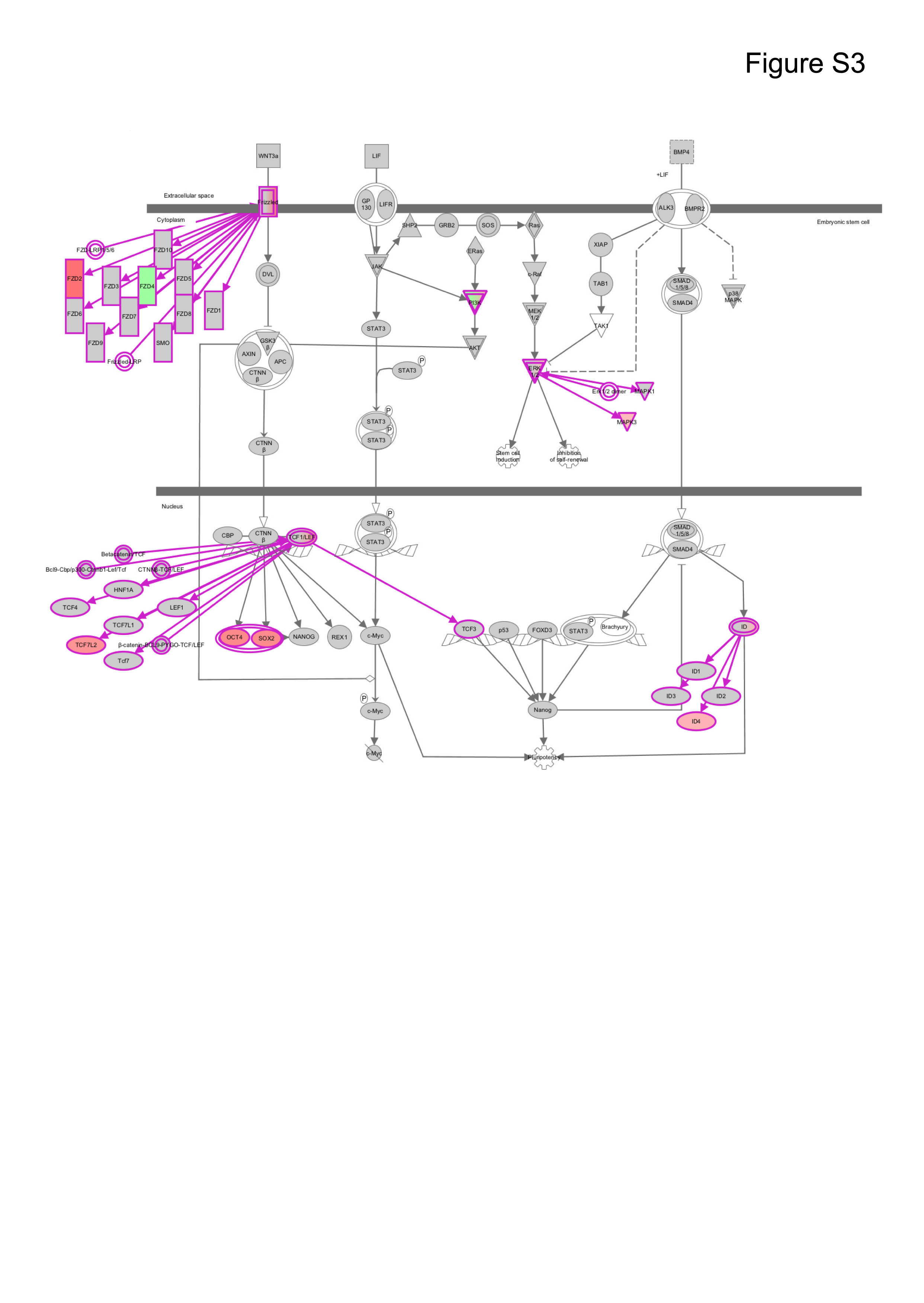

Supplement: S3 Fig — DEGs were subjected to PCA analysis to illustrate the relationships among transcriptomes. First, the first principal component (PC1) captured the differences between neonatal and adult germ cells, suggesting considerable regulatory differences in neonatal versus adult spermatogonia. Second, neonatal KIT- and KIT+ cells clustered closely together, indicating that early germ cells share gene expression properties that commonly define them in transcriptional space despite different differentiation status. Third, KIT- and KIT+ cells are well separated from each other in adult spermatogonia, in both normal and aged adults, reflecting that they acquired drastic transcription dynamics upon differentiation compared to the neonatal stage. Lastly, normal adult and aged cells are positioned far away from each other and KIT- cells are more separated compared to KIT+ cells, suggesting that age factor has a more pronounced effect on the undifferentiated spermatogonia than differentiating cells. (TIF) [file pgen.1009369.s003.tif]

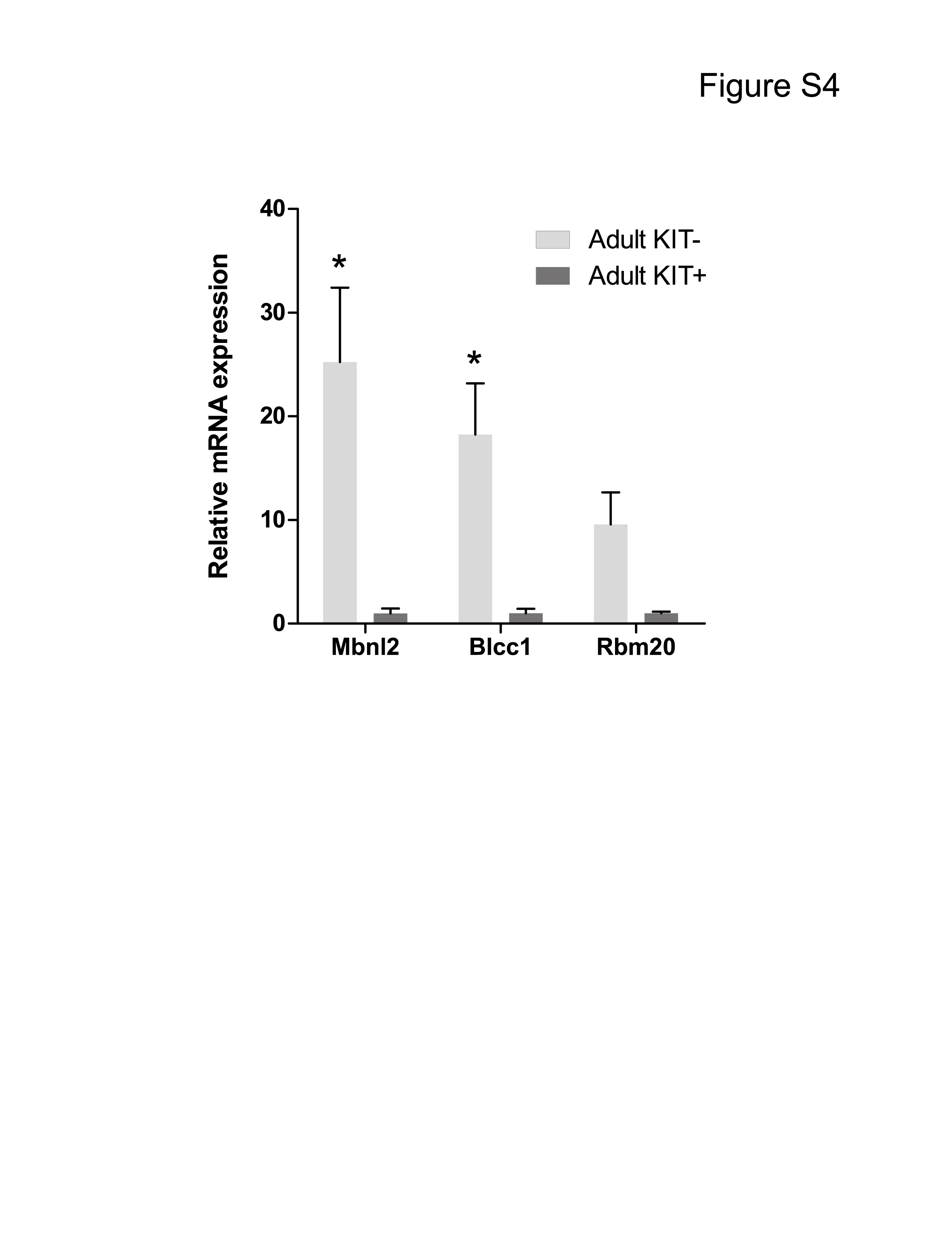

Supplement: S4 Fig — qRT-PCR analysis of expression of splicing factors Mbnl2, Bicc1 and Rbm20 in adult KIT- cells compared with adult KIT+ cells (p < 0.05, n = 2, unpaired t-test). Error bars are plotted with SD. (TIF) [file pgen.1009369.s004.tif]

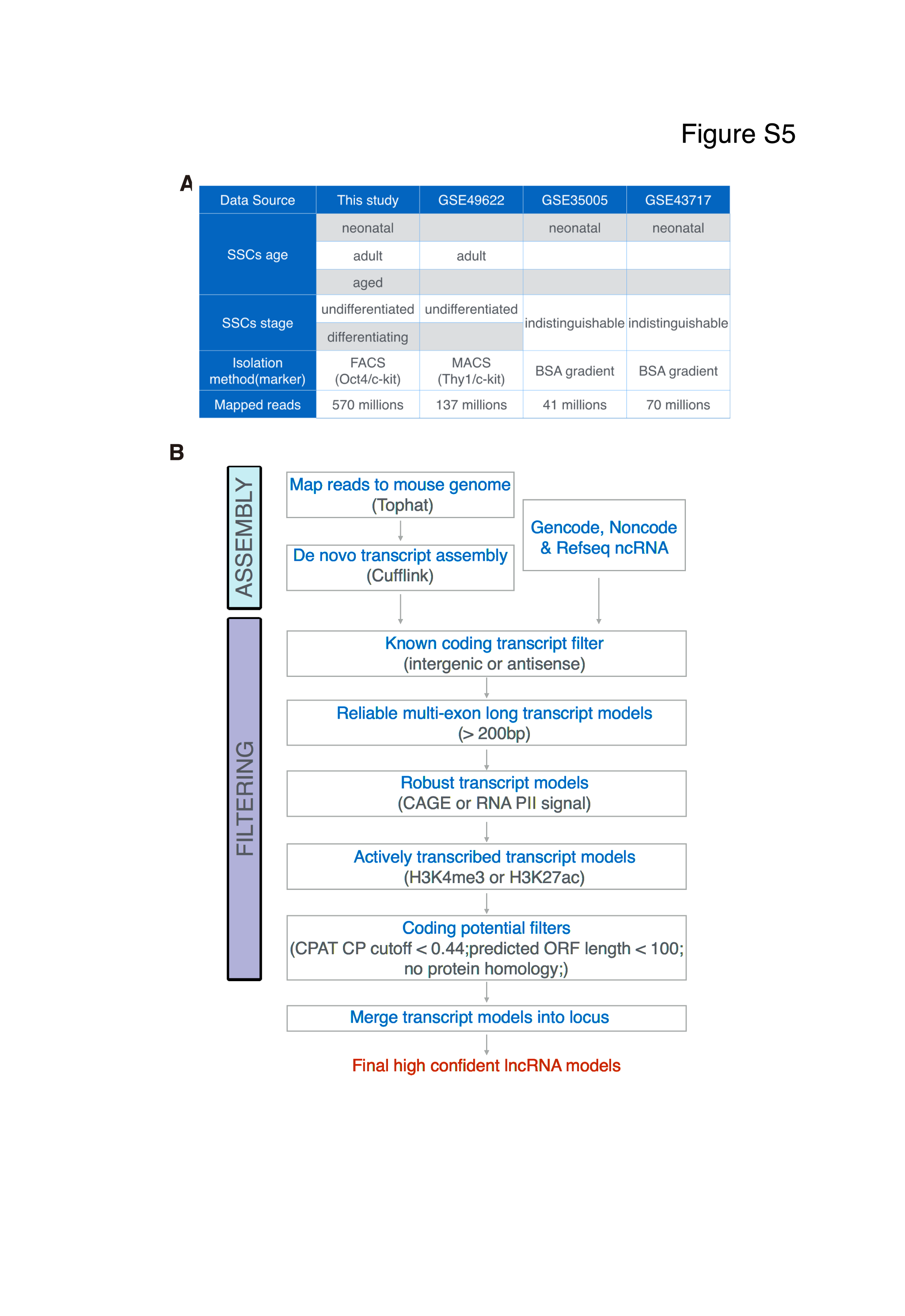

Supplement: S5 Fig — (A) Data sources for novel lncRNA identification. (B) A bioinformatics pipeline for discovery lncRNAs in SSC. See S1 Text Supplemental Methods session for details. Raw reads are first mapped onto the reference mouse genome. The initial assemblies are categorized by cuffcompare, compared with the combined gene annotations. The lncRScan program is performed to detect the novel lncRNAs from the high-quality assemblies according to multiple criteria. (TIF) [file pgen.1009369.s005.tif]

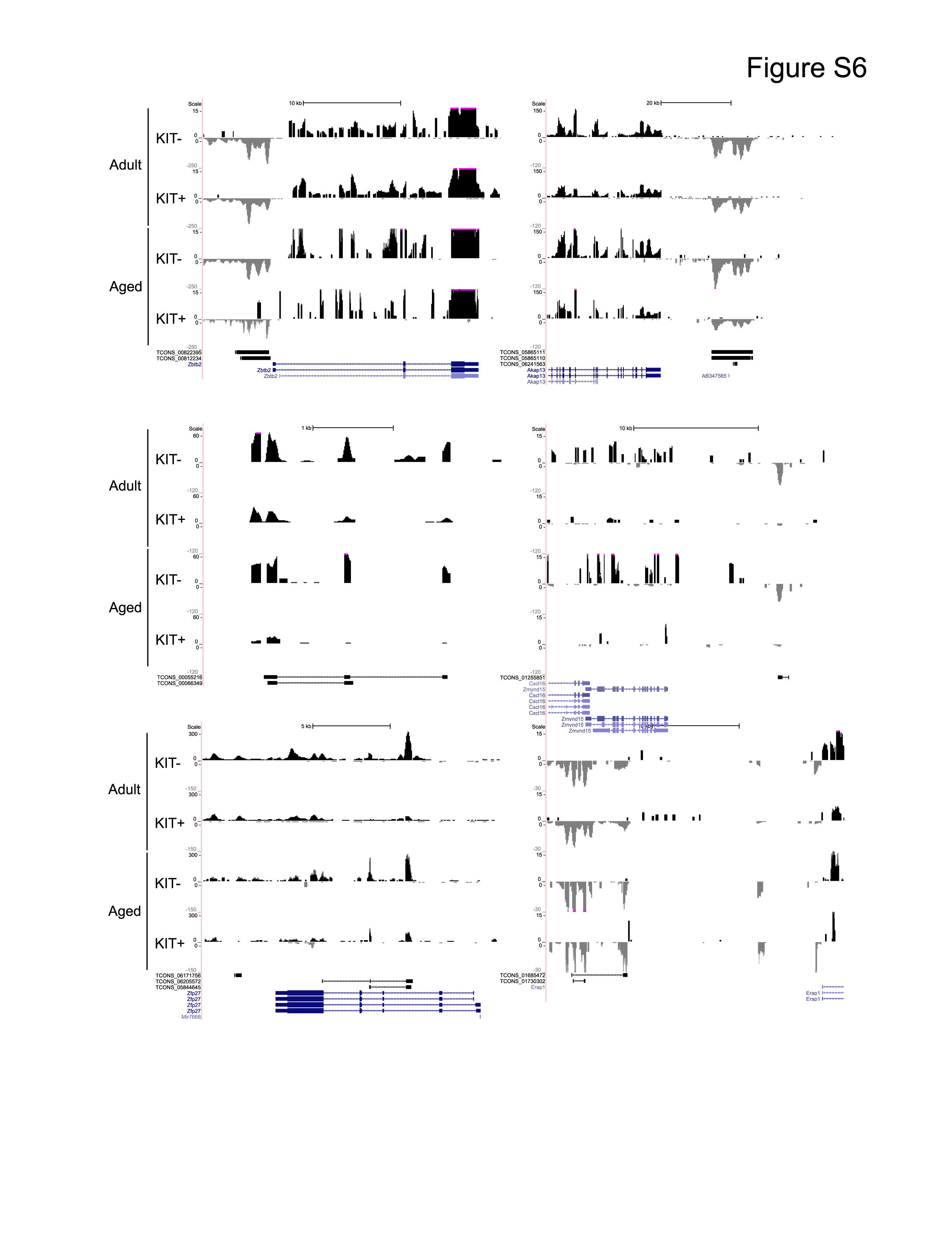

Supplement: S6 Fig — (TIF) [file pgen.1009369.s006.tif]

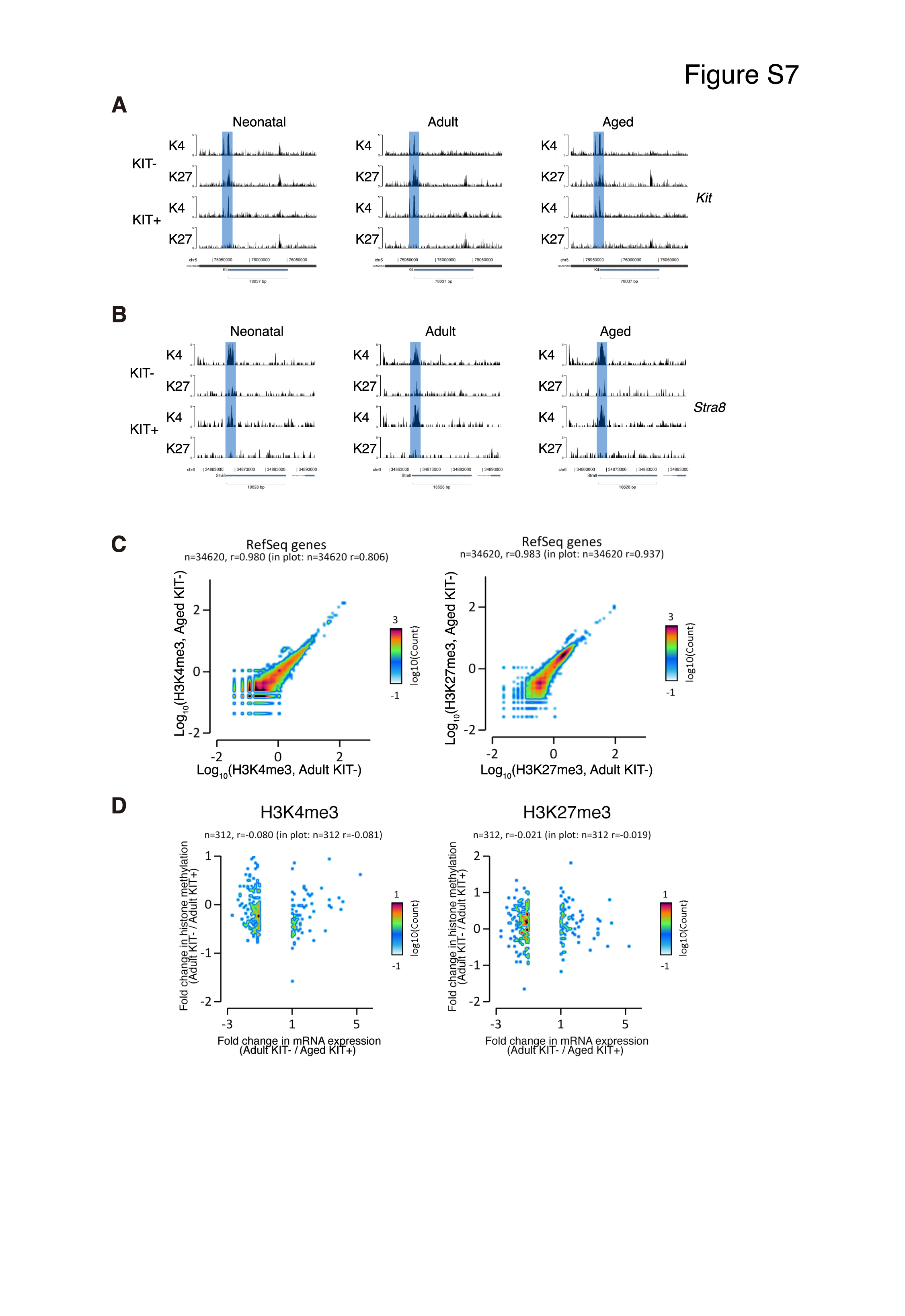

Supplement: S7 Fig — (A) and (B) Genome browser representation of H3K4me3 and H3K27me3 modification at selected genes. (C) Quantitative comparisons of promoter read coverage (reads per kilobase) of each histone modification using 2.5-kb TSS centered bins. (D) Change in the enrichment of each histone modification bound to a gene promoter plotted against the change in expression of that gene using the set of differentially expressed genes during SSC aging. (TIF) [file pgen.1009369.s007.tif]

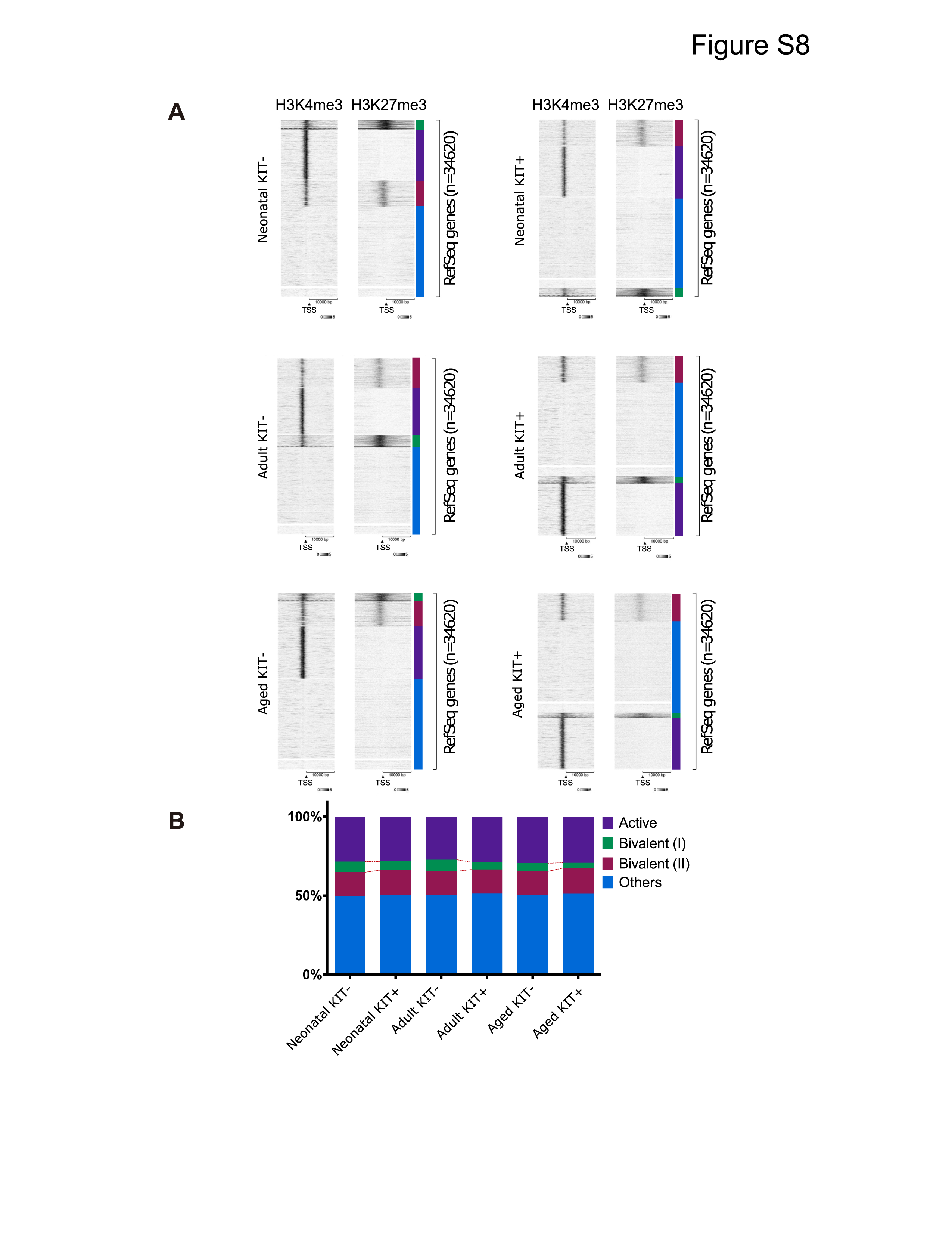

Supplement: S8 Fig — (A) Clustering heatmaps demonstrate the distribution of H3K4me3 and H3K27me3 histone modifications at the promoters of the annotated transcripts in each cell population. (B) Comparison of the number of bivalent promoters in the different cell types according to clustering results showing in (A). (TIF) [file pgen.1009369.s008.tif]

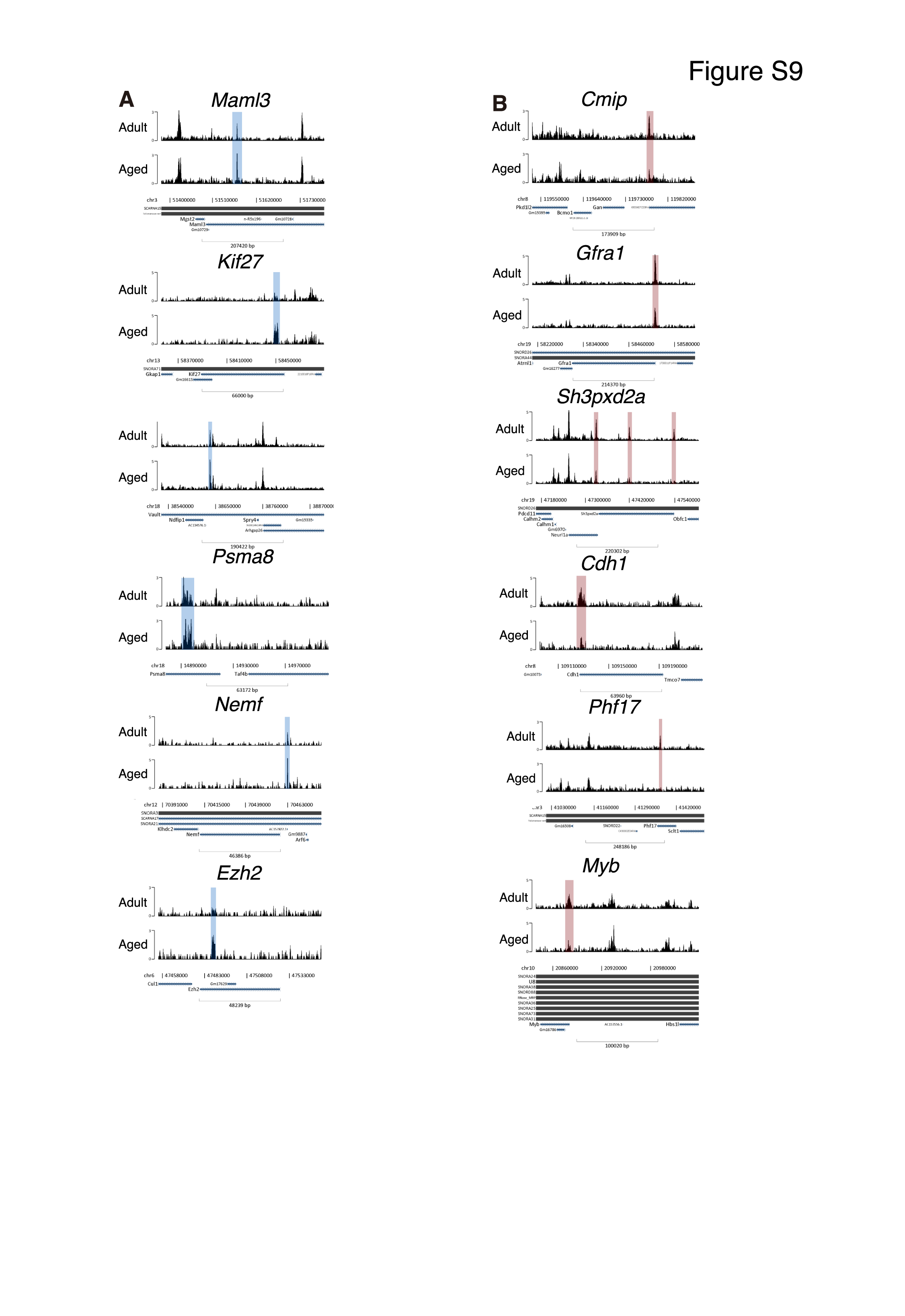

Supplement: S9 Fig — Examples of individual genes showing the increased (A) or decreased (B) H3K27me3 enrichment. (TIF) [file pgen.1009369.s009.tif]
