# Supplementary material for: Transcriptomic and epigenomic profiling of young and aged spermatogonial stem cells reveals molecular targets regulating differentiation
Source: PLoS Genet. 2021 Jul 8;17(7):e1009369. doi: 10.1371/journal.pgen.1009369 (PMC8291634; doi:10.1371/journal.pgen.1009369)
Supplement: S1 Text — (DOCX) [file pgen.1009369.s010.docx]

# SUPPLEMENTAL EXPERIMENTAL PROCEDURES

## Immunohistochemistry for whole mount tubules and imaging

Seminiferous tubules were decapsulated from mouse testes and gently pulled apart with a pair of fine forceps in petri dishes. The resulting tubules were then fixed in 4% paraformaldehyde solution for 2 hours followed by permeabilization with 0.2% Triton-X and dehydration in a graded series of methanol washes. After rehydration in PBS, tubules were processed with staining protocol. Briefly, sections were permeabilized in PBS with 0.2% Triton-X and blocked with PBS supplemented with 0.2% Triton X-100 and 10% normal donkey serum (Jackson ImmunoResearch) for 1 hour at room temperature, followed by incubation with primary antibody overnight at 4 °C. After washing in PBS, sections were incubated with corresponding fluorescence-conjugated secondary antibody. Finally, the slides were washed with PBS and mounted with PermaFluor aqueous mounting medium (Thermo Fisher). Primary and secondary antibodies utilized in this study are listed in Table 1. Fluorescent microscopy and white field images were acquired using a Leica SP2 confocal microscope. All images were captured using Leica Application Suite Software.

## Isolation of undifferentiated and differentiating spermatogonia by fluorescence-activated cell sorting (FACS)

Testicular cells from adult and aged Oct4-GFP mice were isolated by a two-step enzymatic digestion. Firstly, the isolated tubules were incubated in RPMI with 1 mg/ml type 2 collagenase (Worthington) and 5μg/ml DNase I (Sigma-Aldrich) at 37°C for 20 minutes with occasional shaking. After several washes by sedimentation in RPMI, tubular fragments were digested in RPMI with 1 mg/ml type 2 collagenase (Worthington), 1 mg/ml hyaluronidase (Sigma-Aldrich) and 5μg/ml DNase I (Sigma-Aldrich) at 37°C for 30 minutes with occasional shaking. Single-cell suspensions were obtained by passing through a cell strainer (40μm). The resulting cell suspensions were directly separated by centrifugation in 27% Percoll to concentrate spermatogonia and to reduce cellular debris. Spermatogonia-enriched bottom fraction was collected and washed followed by processing to antibody staining. After incubation with an antibody against KIT at 4°C for 30 minutes, cell fractions were collected with a FACS Aria II cell sorter (BD Biosciences). Age and strain matched non-reporter mice i.e. C57BL/6 mice were used for cell purification as negative control devoid of GFP expression for cell sorting experiments. Positive antibody labeling was determined by comparison to staining with isotype control antibodies.

## RNA-Seq

1x10^6^ primary cells of each population in two independent experiments were FACS-sorted as described above. Total RNA was isolated from each cell fraction using the AllPrep DNA/RNA Mini kit (Qiagen, Valencia, CA, USA) according to the manufacturer's instructions. RNA yield and purity were evaluated using a NanoDrop 1000 spectrophotometer (NanoDrop Technologies/Thermo Scientific, Waltham, MA). Quality and integrity were determined by RNA 6000 Nano kit with a Bioanalyzer 2100 (Agilent Technologies, Santa Clara, CA). The ratio of absorbance at 260 and 280 nm was ≥1.9 and the RNA integrity number (RIN) was >9 for all samples. Sequencing libraries were prepared with ribosomal RNA (rRNA) depletion using a Ribo-Zero Gold kit (Epicentre) followed by Apollo 324 NGS Library Prep System (WaferGen Biosystems, Fremont, CA, USA). Two biological replicates of the prepared libraries were sequenced on an Illumina HiSeq 2000 (Illumina, San Diego, CA) with 100 base pairs (bp) paired-end RNA-Seq reads.

## Data analysis of bulk RNA-Seq experiments

Raw reads were pre-processed with Trimmomatic for Illumina adaptor sequences trimming and mapped to mouse (mm9) genome. Counts were assigned to genes defined by the Ensembl (release 67) annotation using featureCount R function. Differentially expressed genes were identified with the DESeq2 package [[1]](https://sciwheel.com/work/citation?ids=129353&pre=&suf=&sa=0) using the cutoffs of FDR ≤0.05 and fold change of 1.5. Differential exon usage analysis was performed using DEXSeq with default setting [[2]](https://sciwheel.com/work/citation?ids=394516&pre=&suf=&sa=0).

## Functional Annotation of Differentially Expressed Genes by IPA

To define the functional networks of differentially expressed genes, data was analyzed by using the Ingenuity Pathway Analysis (IPA, Ingenuity Systems) that calculates a significance score (network score) for each network. A data set containing the gene identifiers and their corresponding fold change (log2) values were uploaded into the IPA software. Each gene identifier was mapped to its corresponding gene object in the Ingenuity Pathways Knowledge Base to identify molecules whose expression was significantly differentially regulated (focus genes or Networks Eligible molecules). These focus genes were overlaid onto a global molecular network developed from information contained in the Ingenuity Knowledge Base. Networks of these focus genes were then algorithmically generated based on their connectivity. A network is a graphical representation of the molecular relationships between genes or gene products, which are represented as nodes, and the biological relationship between two nodes is represented as an edge (line). All edges are supported by at least one reference from the literature, or from canonical information stored in the Ingenuity Pathways Knowledge Base.

## Identification and quantification of lncRNA

As a basis for our analysis of lncRNA expression, we utilized our high quality, ribosome RNA-depleted RNA-Seq dataset. We also included a poly(A)+ RNA-Seq dataset obtained from THY1+/KIT- cells that were isolated from adult mice [[3]](https://sciwheel.com/work/citation?ids=2297742&pre=&suf=&sa=0). and additional two RNA-Seq datasets obtained from BSA gradient enriched neonatal spermatogonia [[4,5]](https://sciwheel.com/work/citation?ids=484537,1009837&pre=&pre=&suf=&suf=&sa=0,0). Together, we combined a total of ~1 billion uniquely mapping sequencing reads to survey lncRNA expression in SSC (S3A Fig).

We employed *ab initio* transcript assembly to assess the completeness of the known annotation about lncRNAs expressed in SSCs. Raw reads were mapped to the mouse mm9 reference genome using TopHat and *de novo* transcriptome assembly of mapped reads was performed using Cufflinks. Gencode, Noncode and RefSeq annotated transcripts were merged into one set of gene annotation, and these annotated transcripts were filtered out from Cufflinks-assembled transcriptomes. The novel transcripts were then categorized into different categories according to their locations compared with the reference genes. To exclude artifacts of the sequence alignment process, unspliced intronic pre-mRNA, and genomic DNA contamination, only lncRNAs transcribed within intergenic and non-exon-overlapping regions were retained. We further filtered the list of novel transcripts by CAGE signaling, RNA PolII binding and H3K4me3 enrichment, and determined the Coding Potential Calculator (CPC) score of each transcript with default setting. Finally, overlapping lncRNA loci were merged to create a single 'consensus' transcript model.

## ChIP-Seq library preparation and sequencing

ChIP experiments and ChIP-Seq libraries were prepared using Diagenode True MicroChIP kit according to the manufacturer's protocol. 2x10^5^ freshly sorted Oct4-GFP+/KIT- or Oct4-GFP+/KIT+ cells were cross-linked with 1% formaldehyde for 10 minutes. The crosslinking was stopped by incubation with 0.125M glycine at room temperature for 5 minutes. Chromatin was fragmented to 200 to 600 bp by sonication using a Bioruptor sonicator (Diagenode, Liège, Belgium). 1ug antibodies against H3K4me3 or H3K27me3 were used for ChIP. Half of the chromatin was used for each IP, and 10% of the chromatin solution was reserved as input control. The chromatin immunoprecipitated (ChIPed) DNA was subjected to library preparation using MicroPlex Library Preparation kit (Diagenode, Liège, Belgium). The prepared library was sequenced with single-end 50bp reads using the Illumina HiSeq 2000.

## ChIP-Seq data analysis

Reads were mapped to the mouse genome (mm9 assembly) using Bowtie [[6]](https://sciwheel.com/work/citation?ids=48646&pre=&suf=&sa=0). Mapped reads were filtered for PCR duplicates using MACS2 [[7]](https://sciwheel.com/work/citation?ids=57981&pre=&suf=&sa=0). Peak calling and visualization of tracks were done using EaSeq with default setting [[8]](https://sciwheel.com/work/citation?ids=1308082&pre=&suf=&sa=0) and heat maps were made using EaSeq or seqMINER [[9]](https://sciwheel.com/work/citation?ids=49894&pre=&suf=&sa=0). In our annotation, a TSS region is defined as ±2.5 kb relative to the TSS. For density plots, we generated bigwig files allowing only one read per chromosomal position, eliminating potential spurious spikes. For density plots, the regions of ±5 kb for all annotated TSSs were divided into 100 equal-sized bins.

## Reduced representation bisulfite sequencing (RRBS) and oxRRBS

RRBS libraries from oxidized and non-oxidized DNA were prepared using TrueMethyl Kit according to the manual's instruction (Cambridge Epigenetix). Briefly, 0.5ug of genomic DNA was digested with MspI followed by end repair, A-tailing, and ligation of adaptors. Adaptor-ligated MspI-digested DNA was size-selected (110-380 bp) and purified. 1/5 the DNA was kept for the generation of the non-oxidized library and remaining DNA was subjected to oxidation. Both oxidized and non-oxidized DNA samples were bisulfite-treated. Final library amplification was carried out for 18 cycles, after which the libraries were purified using AMPure XP beads (Agencourt) and sequenced on Illumina platform. The raw reads were quality-checked with FastQC (http://www.bioinformatics.babraham.ac.uk/projects/fastqc/), and low-quality reads and adapters were removed using Trim Galore (http://www.bioinformatics.babraham.ac.uk/projects/trim_galore/) using '--rrbs' trimming mode. Trimmed sequences were mapped to the mouse genome with Bismark [[10]](https://sciwheel.com/work/citation?ids=316455&pre=&suf=&sa=0). The extracted methylation calls were subjected to the subsequent in-depth analysis performed with methyKit packages in R [[11]](https://sciwheel.com/work/citation?ids=27453&pre=&suf=&sa=0). Specifically, the Bismark coverage files were loaded into the methylKit package and the percentage of methylation of individual CpGs was calculated. Bases with high (above 99.9th percentile of coverage in each sample) and low (below 10× coverage, CpG10) read coverage were discarded to prevent PCR bias and increase the power of the statistical tests. 5hmC percentage at each CpG site was computed as the unconverted CpG ratio in RRBS reaction subtracted by the counterpart in oxRRBS reaction of the same DNA sample. Coverage and correlation plots were also generated by methylKit. To quantify the average methylation level within a fixed bin size, we used methylKit to tile the genome with windows of different length and summarize the methylation information on the tiles. Metagene plots of 5mC/5hmC content were generated with SeqPlots software [[12]](https://sciwheel.com/work/citation?ids=2909491&pre=&suf=&sa=0).

## Germ cell culture

Oct4-GFP+/KIT- cells were isolated from 3-month-old Oct4-GFP mice by FACS cell sorting as described above. Cells were plated at a density of 1.5 to 2 x 10^5^ per well on 12-well plates with mitomycin treated (Sigma-Aldrich) MEFs feeder layers and cultured in a serum-free medium consisting of StemPro-34 SFM medium supplemented with StemPro-34 nutrient supplement (Life Technologies, Carlsbad, CA, USA), 0.2% bovine serum albumin (MP Biochemicals, Santa Ana, CA), 1% fetal bovine serum (embryonic stem cell-qualified, Life Technologies), 50 U/ml penicillin-streptomycin (Life Technologies), 2 mM GlutaMAX (Life Technologies), 1% mM non-essential amino acids (Life Technologies), 1% minimal essential medium (MEM) vitamin solution (Life Technologies), 1 mM sodium pyruvate (Life Technologies), 50 μM 2-mercaptoethanol (Sigma-Aldrich), 25 μg/ml insulin (Sigma-Aldrich), 100 μg/ml transferrin (Sigma-Aldrich), 60 μM putrescine (Sigma-Aldrich), 30 nM sodium selenite (Sigma-Aldrich), 1 mg/ml D-(+)-glucose (Sigma-Aldrich), 1 μl/ml Dl-Lactic acid (Sigma-Aldrich), 60 ng/ml progesterone (Sigma-Aldrich), 30ng/ml β-estradiol (Sigma-Aldrich), 10 μg/ml D-biotin (Sigma-Aldrich), 100 μM ascorbic acid (Sigma-Aldrich), 40 ng/ml human GDNF (R&D Systems, Minneapolis, MN, USA), 20 ng/ml mouse epidermal growth factor (Life Technologies), and 10ng/ml human basic fibroblast growth factor (BD Biosciences, San Jose, CA, USA). Cultures were maintained at 37°C in an incubator with humidified 5% CO2 and 95% air atmosphere. Half of the medium was replaced with fresh medium every 2–3 days and cells were passaged enzymatically using 0.25% trypsin/EDTA (Life Technologies) at a ratio of 1: 2 or 1: 3 every 6 to 7 days onto new mitomycin-treated MEF plates. For RA-induced differentiation, 5mM all-trans-RA (Sigma-Aldrich) stock in ethanol was diluted to 1 μM in medium before applying to cells while vehicle (0.1% ethanol) was added in the control group.

## Gene Ontology (GO) analysis

GO enrichment was performed using DAVID [[13]](https://sciwheel.com/work/citation?ids=43636&pre=&suf=&sa=0), ToppGene Suite [[14]](https://sciwheel.com/work/citation?ids=1399867&pre=&suf=&sa=0). A hypergeometric test with the Benjamini and Hochberg false discovery rate (FDR) was performed using the default parameters to adjust p-value.

# Table 1. List of antibodies.

| **Primary antibody** | **Species reactivity** | **Company** | **Cat. No.** | **Assay** |
| --- | --- | --- | --- | --- |
| KIT | Mouse, Rat, Human | Santa Cruz | sc-1494 | Immunostaining |
| 5hmC | N.A | Active Motif | 39770 | Immunostaining |
| 5fC | N.A | Active Motif | 61224 | Immunostaining |
| 5caC | N.A | Active Motif | 61226 | Immunostaining |
| GFRA1 | Mouse, Rat | R&D | AF560 | Immunostaining |
| PLZF | Human, Mouse, Rat | Santa Cruz | SC-22839 | Immunostaining |
| SYCP3 | Mouse, Rat, Human, Pig | Abcam | ab15093 | Immunostaining |
| SOX9 | Human, Mouse, Rat | Millipore | AB5535 | Immunostaining |
| H3K4me3 | Budding Yeast, Human, Mouse, Wide Range Predicted | Active Motif | 39159 | Immunostaining |
| H3K27me3 | Human, Mouse | Millipore | 07-449 | ChIP-seq |
| APC anti-KIT | Mouse | BD Biosciences | 553356 | FACS |
| **Secondary antibody** | **Species reactivity** | **Company** | **Cat. No.** | **Assay** |
| Donkey anti-Goat IgG, Alexa Fluor 647 | Goat | Invitrogen | A21447 | Immunostaining |
| Donkey anti-Rabbit IgG, Rhodamine Red™-X | Rabbit | Jackson Immunoresearch | 711-295-152 | Immunostaining |
| Donkey anti-Goat-568 | Goat | Thermo Fisher | A11057 | Immunostaining |
| Goat anti-rabbit-568 | Rabbit | Thermo Fisher | A11036 | Immunostaining |

# References:

[1. Love MI, Huber W, Anders S. Moderated estimation of fold change and  dispersion for RNA-seq data with DESeq2. Genome Biol. 2014;15: 550. doi:10.1186/s13059-014-0550-8](https://sciwheel.com/work/bibliography/129353)

[2. Anders S, Reyes A, Huber W. Detecting differential usage of exons from RNA-seq data. Genome Res. 2012;22: 2008–2017. doi:10.1101/gr.133744.111](https://sciwheel.com/work/bibliography/394516)

[3. Hammoud SS, Low DHP, Yi C, Carrell DT, Guccione E, Cairns BR. Chromatin and transcription transitions of mammalian adult germline stem cells and spermatogenesis. Cell Stem Cell. 2014;15: 239–253. doi:10.1016/j.stem.2014.04.006](https://sciwheel.com/work/bibliography/2297742)

[4. Soumillon M, Necsulea A, Weier M, Brawand D, Zhang X, Gu H, et al. Cellular source and mechanisms of high transcriptome complexity in the mammalian testis. Cell Rep. 2013;3: 2179–2190. doi:10.1016/j.celrep.2013.05.031](https://sciwheel.com/work/bibliography/484537)

[5. Gan H, Wen L, Liao S, Lin X, Ma T, Liu J, et al. Dynamics of 5-hydroxymethylcytosine during mouse spermatogenesis. Nat Commun. 2013;4: 1995. doi:10.1038/ncomms2995](https://sciwheel.com/work/bibliography/1009837)

[6. Langmead B, Trapnell C, Pop M, Salzberg SL. Ultrafast and memory-efficient alignment of short DNA sequences to the human genome. Genome Biol. 2009;10: R25. doi:10.1186/gb-2009-10-3-r25](https://sciwheel.com/work/bibliography/48646)

[7. Zhang Y, Liu T, Meyer CA, Eeckhoute J, Johnson DS, Bernstein BE, et al. Model-based analysis of ChIP-Seq (MACS). Genome Biol. 2008;9: R137. doi:10.1186/gb-2008-9-9-r137](https://sciwheel.com/work/bibliography/57981)

[8. Lerdrup M, Johansen JV, Agrawal-Singh S, Hansen K. An interactive environment for agile analysis and visualization of ChIP-sequencing data. Nat Struct Mol Biol. 2016;23: 349–357. doi:10.1038/nsmb.3180](https://sciwheel.com/work/bibliography/1308082)

[9. Ye T, Krebs AR, Choukrallah M-A, Keime C, Plewniak F, Davidson I, et al. seqMINER: an integrated ChIP-seq data interpretation platform. Nucleic Acids Res. 2011;39: e35. doi:10.1093/nar/gkq1287](https://sciwheel.com/work/bibliography/49894)

[10. Krueger F, Andrews SR. Bismark: a flexible aligner and methylation caller for Bisulfite-Seq applications. Bioinformatics. 2011;27: 1571–1572. doi:10.1093/bioinformatics/btr167](https://sciwheel.com/work/bibliography/316455)

[11. Akalin A, Kormaksson M, Li S, Garrett-Bakelman FE, Figueroa ME, Melnick A, et al. methylKit: a comprehensive R package for the analysis of genome-wide DNA methylation profiles. Genome Biol. 2012;13: R87. doi:10.1186/gb-2012-13-10-r87](https://sciwheel.com/work/bibliography/27453)

[12. Stempor P, Ahringer J. SeqPlots - Interactive software for exploratory data analyses, pattern discovery and visualization in genomics. [version 1; peer review: 2 approved, 1 approved with reservations]. Wellcome Open Res. 2016;1: 14. doi:10.12688/wellcomeopenres.10004.1](https://sciwheel.com/work/bibliography/2909491)

[13. Huang DW, Sherman BT, Lempicki RA. Systematic and integrative analysis of large gene lists using DAVID bioinformatics resources. Nat Protoc. 2009;4: 44–57. doi:10.1038/nprot.2008.211](https://sciwheel.com/work/bibliography/43636)

[14. Chen J, Bardes EE, Aronow BJ, Jegga AG. ToppGene Suite for gene list enrichment analysis and candidate gene prioritization. Nucleic Acids Res. 2009;37: W305-11. doi:10.1093/nar/gkp427](https://sciwheel.com/work/bibliography/1399867)
